# Predictors of Environmental Sensitivity in Syrian refugee children

**DOI:** 10.1111/jcpp.14178

**Published:** 2025-06-10

**Authors:** Andrew K. May, Demelza Smeeth, Fiona McEwen, Elie Karam, Michael Pluess

**Affiliations:** ^1^ Department of Psychological Sciences, School of Psychology University of Surrey Guildford UK; ^2^ Department of Biomolecular Sciences, School of Life Science, Pharmacy, and Chemistry Kingston University London London UK; ^3^ Biological and Experimental Psychology, School of Biological and Behavioural Sciences Queen Mary University of London London UK; ^4^ Department of War Studies King's College London London UK; ^5^ Department of Psychiatry and Clinical Psychology, Saint George Hospital University Medical Center Saint George University of Beirut, Institute for Development, Research, Advocacy and Applied Care (IDRAAC) Beirut Lebanon

**Keywords:** Environmental Sensitivity, predictors, refugees, children, Syria

## Abstract

**Background:**

Although more prone to psychopathology on average, refugee children differ in their response to adversity. Growing evidence attributes some of these individual differences to varying levels of Environmental Sensitivity – the extent to which children perceive and process contextual influences. However, there is limited knowledge of how Environmental Sensitivity is developmentally influenced, particularly in the refugee setting.

**Methods:**

Here, we investigated whether individual‐, family‐ and community‐level predictors (psychosocial and genetic) were associated with self‐reported Environmental Sensitivity and its subscales (measured using the 12‐item Highly Sensitive Child Scale). Participants were a subsample (*n* = 1,409) from a cohort of Syrian refugee children and their biological mothers, recruited from informal tented settlements in Lebanon. Multivariate adaptive regression spline models were fitted to identify the best selection from over 40 available predictors.

**Results:**

Twelve predictors of Environmental Sensitivity emerged, with the five most commonly selected being maternal behavioural control, human insecurity, positive home experiences, maternal anxiety and child‐reported child abuse, the latter three of which were also suggested to predict changes in sensitivity over a 12‐month period. Some predictors such as maternal PTSD, war exposure and bullying showed a non‐linear, V‐shape relationship with sensitivity. All effect sizes, however, were small.

**Conclusions:**

Our findings suggest that both highly supportive and highly adverse contextual factors associate with greater childhood Environmental Sensitivity, in line with current theorising. Despite previous suggestive evidence, we did not find that polygenic scores for autism and attention deficit hyperactivity disorder predicted sensitivity. Further research into predictors of Environmental Sensitivity is encouraged, as this may help with improved assessment of the trait in children.

## Introduction

Refugee children often endure numerous traumatic experiences, elevating their risk for mental health problems (Eruyar, Maltby, & Vostanis, 2018). However, prevalence estimates for psychopathology vary (McEwen et al., 2023), and many refugee children appear resilient (Dangmann, Dybdahl, & Solberg, 2022), suggesting that children differ in their response to adversity. Part of this variability may be attributable to differences in *Environmental Sensitivity* (Karam et al., 2024) – the extent to which children detect and are impacted by their environmental exposures. About 50% of the variance in sensitivity is determined by environmental influences (Assary, Zavos, Krapohl, Keers, & Pluess, 2021), but knowledge of the specific factors that shape sensitivity remains limited. Here we attempt to identify predictors of Environmental Sensitivity from individual, family, and community variables among Syrian refugee children in Lebanon.

### The Environmental Sensitivity framework

Inter‐individual differences in how children perceive and process environmental influences have been acknowledged across academic fields (Belsky, 2016). To account for these differences, several theories based on psychological (Aron, Aron, & Jagiellowicz, 2012), physiological (Boyce & Ellis, 2005) or genetic arguments (Belsky & Pluess, 2009) have been integrated into the *Environmental Sensitivity (ES)* framework (Pluess, 2015). In brief, ES contends that the different capacities individuals have to register and process environmental information can be quantified along a continuum, based on functional variability in the central and peripheral nervous systems arising from normal genetic variation and gene‐x‐environment interplay (Pluess, Lionetti, Aron, & Aron, 2023). Accordingly, imaging studies have revealed differences in brain region activation between those high and low in ES (Acevedo et al., 2014; Acevedo, Aron, Pospos, & Jessen, 2018; Acevedo, Jagiellowicz, Aron, Marhenke, & Aron, 2017). Meanwhile, a twin study suggests that genetic and environmental influences contribute equally to the development of sensitivity (Assary et al., 2021), and genetic influences on autism (Assary et al., 2024) and ADHD (Weyn et al., 2025) appear to overlap with ES.

A central claim of ES theorising is that heightened sensitivity renders children disproportionately receptive to both positive *and* negative environmental factors (Boyce, 2015). This ‘for better and for worse’ pattern of influence is supported by numerous studies (Belsky & Bakermans‐Kranenburg, 2007; Rabinowitz & Drabick, 2017) – compared to less sensitive peers, highly sensitive children respond more strongly to negative feedback (Slagt, Dubas, van Aken, Ellis, & Deković, 2017), hostile parenting (Slagt, Dubas, Ellis, Van Aken, & Deković, 2019), and deprived material backgrounds (Keers & Pluess, 2017) but also benefit disproportionately from positive school (Iimura & Kibe, 2020), depression (Pluess & Boniwell, 2015), and anti‐bullying interventions (Nocentini, Menesini, & Pluess, 2018).

### Environmental Sensitivity in refugees

‘For better and for worse’ outcomes have also been suggested for refugee children. On balance, highly sensitive refugee children appear at greater risk for mental illness (May, Smeeth, McEwen, Karam, Rieder, et al., 2024) due to an increased vulnerability to trauma. This risk is potentially magnified in sensitive children without a history of adversity (Karam et al., 2019). However, positive home experiences may buffer sensitive children against mental illness, more so than less sensitive children (Karam et al., 2024). Although few in number, these studies reiterate the potential clinical utility of ES (Belsky & van Ijzendoorn, 2015), particularly in the refugee context, assuming ES can be reliably measured.

### Predictors of Environmental Sensitivity

Currently, ES is measured using the Highly Sensitive Child Scale, which is prone to self‐report issues, including response bias, cross‐cultural variance, comprehension differences, and contextual influences (Pluess et al., 2018; Weyn et al., 2019). Such issues could be mitigated by corroborating scale scores with objective measures of genetic and environmental predictors, many of which have been suggested but rarely empirically investigated. Theoretical arguments propose that sensitivity is calibrated by ‘environmental forecasting’ during child rearing (Pluess & Belsky, 2011). According to Biological Sensitivity to Context (Boyce & Ellis, 2005), both low and high levels of early adversity may engender greater stress reactivity and thus heightened sensitivity in children. For example, Li, Sturge‐Apple, and Davies (2021) noted that, in a sample of 235 toddlers, ES levels stayed constant over 1 year when environmental harshness (a composite measure of family income, maternal harsh discipline, and maternal sensitivity) was scored as either proportionately high or low. However, intermediate levels of harshness predicted decreases in sensitivity. Whether similar findings extend to refugee children, however, is unknown.

### The present study

The aim of our study was to identify predictors of ES in a cohort of Syrian refugee children (*n* = 1,600, aged 6–20 years old). We regressed self‐reported ES on to environmental (refugee setting, home life, parental quality, bullying, war exposure) and genetic (polygenic scores for sensitivity‐related traits including attention deficit hyperactivity disorder and autism) factors. Non‐parametric and penalised regression models were trained and tested to illuminate the best selection of predictors. Based on existing research, we hypothesised that because children embedded in harsher refugee contexts were more likely to exhibit heightened sensitivity – consonant with Biological Sensitivity to Context (Boyce & Ellis, 2005) – variables associated with adversity would be prioritised in predictor selection. We further hypothesised, based on previous evidence (Assary et al., 2024), that polygenic scores for sensitivity‐related phenotypes would also emerge as predictors of ES, reflecting common genetic underpinnings. The study was preregistered at PsychArchives (May, Smeeth, McEwen, Karam, & Pluess, 2024).

## Methods

### Study participants

Participants were drawn from a cohort of Syrian refugee children and adolescents (*n* = 1,600), and their accompanying caregivers, recruited as part of the biological pathways of risk and resilience (BIOPATH) study, detailed elsewhere (McEwen et al., 2022). Children were eligible to participate provided they had a suitable caregiver, were between the ages of 8 and 16 (to the best of their available knowledge) and had not fled Syria more than four years prior to baseline data collection. Participants were then followed up 1 year later (Wave 2). To improve uniformity in our sub‐sample, we excluded children ineligible for the study (*n* = 6) due to data capture and other logistical errors, children whose participating caregiver was not their biological mother (*n* = 171) and children who reported on the caregiving quality of someone other than their biological mother (*n* = 14). The final sub‐sample comprised 1,409 children at Wave 1 (52% female), with a mean age of 11 years old (Table 1), of which 898 were followed up at Wave 2.

**Table 1:** Descriptive statistics for the study cohort

Characteristic	Wave 1	Wave 2
	*N* = 1,409^a^	*N* = 898^a^
Child age (years)	11.30 (2.38)	12.10 (2.33)
Sex		
Females	735 (52.16%)	480 (53.45%)
Males	674 (47.84%)	418 (46.55%)
Child HSC (total)	5.03 (1.01)	4.79 (0.96)
Child HSC (EOE)	4.73 (1.40)	4.42 (1.43)
Child HSC (LST)	5.00 (1.62)	4.84 (1.66)
Child HSC (AES)	5.42 (1.10)	5.21 (1.19)
PGS000327	10.83 (5.23)	10.83 (5.23)
PGS002790	0.17 (0.09)	0.17 (0.09)
PGS002746	227.13 (169.29)	227.13 (169.29)
PGS003753	9.18 (5.68)	9.18 (5.68)
Mother age (years)	38.51 (7.38)	39.29 (7.62)
Highest level of mother's education		
Did not attend school	812.00 (57.79%)	509.00 (57.19%)
Up to Grade 6	462.00 (32.88%)	298.00 (33.48%)
Up to Grade 9	109.00 (7.76%)	69.00 (7.75%)
Up to Grade 12	22.00 (1.57%)	14.00 (1.57%)
Number of children	5.97 (2.43)	5.97 (2.43)
Birth order of participating child	3.34 (2.50)	3.34 (2.50)
Maternal HSP (total)	5.29 (0.89)	5.20 (0.87)
Maternal PTSD	33.63 (17.83)	24.33 (18.21)
Maternal anxiety	8.22 (5.34)	6.87 (5.33)
Maternal depression	15.38 (6.44)	13.95 (7.39)
Maternal impulsivity	24.24 (5.76)	22.24 (6.18)
Mental health in the past 12 months (MH1)	4.32 (0.82)	4.00 (0.95)
Mental health since onset of war, but prior to the past 12 months (MH2)	4.68 (0.62)	4.56 (0.75)
Mental health prior to the onset of war (MH3)	2.12 (0.95)	1.87 (0.88)
Total abuse (child report)	11.81 (12.10)	8.42 (10.29)
Psychological control	11.21 (2.57)	10.66 (2.20)
Acceptance	27.27 (3.93)	27.53 (4.21)
Behavioural control	13.97 (2.02)	14.08 (2.01)
Mother–child conflict	6.01 (3.17)	7.28 (3.90)
Positive home experiences (PHE)	24.66 (4.62)	24.87 (4.51)
Physical abuse (mother report)	9.05 (6.82)	7.05 (6.56)
Perceived refugee environment	3.21 (0.51)	3.32 (0.49)
Human insecurity	3.69 (0.40)	3.74 (0.36)
Perceived social support	5.56 (0.93)	5.74 (0.99)
Bullying	3.96 (5.13)	3.98 (6.37)
Violent victimisation	0.64 (1.61)	0.40 (1.34)
Collective efficacy and informal social control	31.87 (6.52)	31.55 (7.96)
War exposure	9.60 (5.50)	7.76 (6.05)
Time since leaving Syria		
Less than 1 year ago	262.00 (18.67%)	135.00 (15.03%)
1–2 years ago	201.00 (14.33%)	120.00 (13.36%)
2–3 years ago	187.00 (13.33%)	119.00 (13.25%)
3–4 years ago	527.00 (37.56%)	299.00 (33.30%)
More than 4 years ago	226.00 (16.11%)	225.00 (25.06%)

^a^ Mean (*SD*); *n* (%).

### Ethical considerations

Ethical approval for the study was obtained from the institutional review board at the University of Balamand/Saint George Hospital University Medical Center, Lebanon (ref: IRB/O/024‐16/1815), the Lebanese National Consultative Committee on Ethics, and the Ministry of Public Health in Lebanon.

### Procedure

Child‐caregiver dyads completed a battery of questionnaires capturing demographic and psychosocial information. Measures were selected to assess variables across different ecological strata (Bronfenbrenner, 1979), including individual, family, and community systems.

Instruments without an existing translation were translated into Arabic following a published protocol (McEwen et al., 2021), and piloted within the same target population. A full description of the measures used in the BIOPATH study is available in the supplementary materials of McEwen et al. (2022). Variables selected for the current study are outlined below.

Children also supplied a saliva sample for DNA extraction and genotype analysis. Genotyping, imputation and quality control procedures are detailed in May, Smeeth, McEwen, Karam, Rieder, et al. (2024). Polygenic scores were calculated by means of the Michigan Imputation Server's Polygenic Scores service (Beta version 2.0.0; https://imputationserver.sph.umich.edu).

### Outcome variable

Environmental sensitivity was captured using the 12‐item self‐report Highly Sensitive Child scale (Pluess et al., 2018), scored on a Likert scale from 1 to 7, with higher scores indicating greater sensitivity. While the scale mostly taps behaviours reflecting immediate sensory reactivity in children (e.g. *I love nice tastes*, *I love nice smells*), it has been previously used to index children's responses to environmental adversity (Karam et al., 2024; Scrimin, Osler, Pozzoli, & Moscardino, 2018), as a moderator of intervention effects (Nocentini et al., 2018), and as a marker of vantage sensitivity (Iimura & Kibe, 2020). The instrument has good internal consistency (*α* = .78) and test–retest reliability over 15 days (*r* = .68). Instrument reliability in the BIOPATH sample was comparable but slightly lower (Cronbach's *α* = .73 for wave 1; .62 for Wave 2). The instrument can be subscaled to represent three factors, namely Ease of Excitation (EOE), Low Sensory Threshold (LST) and Aesthetic Sensitivity (AES), with *α* reliabilities at Wave 1 in the current sample of .70, .54 and .45 respectively. Sub‐scale reliabilities are lower in the child scale due to the smaller pool of items.

### Predictor variables

#### Individual‐level predictors

Individual‐level predictors included child age, sex, and four polygenic scores for two sensitivity‐related phenotypes, namely attention deficit hyperactivity disorder (ADHD; PGS002746 and PGS003753) and autism spectrum disorder (ASD; PGS000327 and PGS002790). Coverage within the imputed BIOPATH data was adequate, ranging from 80% to 100%, although all four scores were derived from almost exclusively European ancestry populations (Table S1).

#### Family‐level predictors

Most selected variables assessed features of the family environment, particularly maternal mental health and parenting. These included child‐reported child abuse and neglect (ISPCAN Child Abuse Screening Tool, Runyan, Dunne, & Zolotor, 2009), psychological control (Psychological Control – Disrespect Scale, Barber, Xia, Olsen, McNeely, & Bose, 2012), acceptance (Acceptance subscale of the Child Report of Parent Behavior Inventory, Schaefer, 1965), behavioural control (Brown, Mounts, Lamborn, & Steinberg, 1993), mother–child conflict (Barber, 1999), and positive home experiences (developed by the Institute for Development, Research, Advocacy and Applied Care to measure social context in children affected by war in Lebanon). Caregiver‐reported variables included age, number of children, birth order of participating child, highest level of education, maternal environmental sensitivity (Highly Sensitive Person scale, Pluess et al., 2023), child abuse and neglect (ISPCAN Child Abuse Screening Tool), maternal depression (Center for Epidemiological Studies Short Depression Scale, Radloff, 1977), maternal anxiety (anxiety items from the Depression, Anxiety and Stress Scale, Henry & Crawford, 2005), maternal post‐traumatic stress disorder (PTSD; the PTSD Checklist for DSM‐5, Blevins, Weathers, Davis, Witte, & Domino, 2015), maternal impulsiveness (Abbreviated Barratt Impulsiveness Scale, Coutlee, Politzer, Hoyle, & Huettel, 2014), perceived stress (Perceived Stress Scale, Cohen, Kamarck, & Mermelstein, 1983), and three single‐item measures (MH1–MH3; Ahmad, Jhajj, Stewart, Burghardt, & Bierman, 2014) of self‐rated mental health, adapted from the World Mental Health Survey Composite International Diagnostic Interview (Kessler & Üstün, 2004).

#### Community‐level predictors

We also investigated variables that assessed the wider family and community environment. These included child reports of peer victimisation (Bullying of Refugee Children, a scale developed for the BIOPATH study) and social support (Multidimensional Scale of Perceived Social Support for Arab American Adolescents, Ramaswamy, Aroian, & Templin, 2009), along with caregiver reports of the refugee environment's quality (Perceived Refugee Environment Index, a scale developed for the BIOPATH study), neighbourhood quality (Collective Efficacy and Informal Social Control, Sampson, Raudenbush, & Earls, 1997), and personal and family insecurity (Human Insecurity Scale, Ziadni et al., 2011). In addition, both child and caregiver completed the War Events Questionnaire (Karam, Al‐Atrash, Saliba, Melhem, & Howard, 1999), and responses were combined into a single measure of the war‐related events witnessed by the dyad.

### Data analysis

Descriptive summaries of participants were prepared and bivariate analyses conducted using the *gtsummary* package (Sjoberg et al., 2021). Zero‐order correlations between variables were diagrammed using network analysis via the *bootnet* (Epskamp, Borsboom, & Fried, 2018) package.

To identify predictors of sensitivity, we used multivariate adaptive regression splines (MARS), with 10‐fold generalised cross‐validation (repeated three times, for 30 total folds) using the *earth* (Milborrow, 2024) package. MARS combines automatic feature selection with linear and non‐linear modelling. We prioritised this technique given the importance of U‐shape relationships in ES theorising (Ellis & Boyce, 2008). We began by splitting wave 1 data into training (*n* = 1,127) and test (*n* = 282) sets using an 80/20 split. We then regressed (a) total HSC scores and (b) HSC subscales onto all other predictors, allowing one knot per predictor and a maximum of 25 predictors in the final model. Average‐item or total scores were used for each psychological instrument, along with any subscales, except where issues of multicollinearity occurred. Missing values per predictor were binary encoded as a separate categorical variable (which were also used as predictor variables to reveal where missingness may have had influence), and then mean‐imputed to maximise the available sample size. The percentage of missing data was less than 2.1% for all predictors except polygenic scores, which were missing for 13.34% (*n* = 188) of participants whose genotyping results did not pass quality control. Categorical variables were one‐hot encoded using the *vtreat* package (Zumel & Mount, 2016). As calculated using the *pwr* package (Champely, 2020), we were 80% powered to detect variables explaining at minimum 1.6% of the variation in sensitivity, assuming a final model with 25 predictors.

Model fit in the training data was compared to the test data based on the root mean square error (RMSE), the *R* ^2^ value, and the mean absolute error (MAE). As follow‐up analyses, we repeated predictor selection using strictly linear approaches, including forward selection and penalised regression (LASSO and elastic net regression via the *glmnet* package; Friedman, Hastie, & Tibshirani, 2010; Tay, Narasimhan, & Hastie, 2023), comparing which features were selected across all methods. We also repeated MARS model fitting after stratifying the cohort by sex, and by age using three age groups with roughly equivalent sample sizes. These age groups were labelled as ‘children’ (younger than 10), ‘pre‐teens’ (10–12 years old) and ‘teenagers’ (13 and older).

Lastly, as an exploratory analysis, we investigated whether changes in predictor variables were associated with changes in environmental sensitivity from Wave 1 to Wave 2. For participants with data available at both waves (*n* = 898), Wave 2 scores were subtracted from Wave 1 scores on all instruments. Following the same process outlined above, data were then split into training (*n* = 718) and test (*n* = 180) sets and changes in total HSC score were regressed onto changes in other scores (along with time invariant variables) using MARS.

## Results

**Figure 1:**
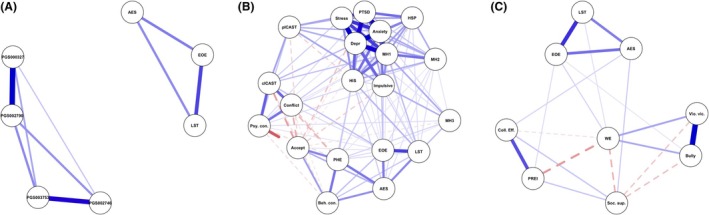
Network diagrams of zero‐order correlations between child sensitivity and variables in different ecological strata at Wave 1. Nodes represent individual variables while edges reflect the nature and strength of correlation. Solid lines indicate positive correlation while dotted lines represent negative correlation. (A) At the individual level, self‐reported sensitivity (LST, AES and EOE) was not associated with polygenic scores for ADHD or ASD. (B) At the family level, self‐reported sensitivity shared strong positive associations with positive home experiences (PHEs), behavioural control (Beh. con.), human insecurity (HIS) and maternal anxiety. (C) At the community level, sensitivity was weakly correlated to war exposure (WE) and the perceived refugee environment (PREI). ADHD, attention deficit hyperactivity disorder; AES, aesthetic sensitivity; ASD, autism spectrum disorder; cICAST, child‐reported child abuse; Depr, maternal depression; EOE, ease of excitation; HSP, maternal sensitivity; LST, low sensory threshold; MH1‐3, single‐item maternal mental health assessment; PGS, polygenic score; pICAST, mother‐reported physical abuse

Descriptive statistics revealed sex differences for several of the psychological instruments (Table 2; Table S2). Zero‐order correlations suggested close associations between child sensitivity and positive home experiences, behavioural control, human insecurity, and maternal anxiety, and modest associations with war exposure and the perceived refugee environment (Figure 1). See Tables S3–S5 for all bivariate correlations.

**Table 2:** Variables differing significantly by sex

Variable	Wave 1				Wave 2			
	Females	Males	*p*‐Value^b^	*q*‐value^c^	Females	Males	*p*‐Value^b^	*q*‐value^c^
	*N* = 735^a^	*N* = 674^a^			*N* = 480^a^	*N* = 418^a^		
Child HSC (total)	5.16 (0.98)	4.89 (1.03)	<.001	**<.001**	4.94 (0.94)	4.61 (0.94)	<.001	**<.001**
Child HSC (EOE)	4.81 (1.40)	4.64 (1.40)	.017	**.017**	4.54 (1.41)	4.29 (1.45)	.015	**.029**
Child HSC (LST)	5.19 (1.52)	4.78 (1.70)	<.001	**<.001**	5.18 (1.57)	4.45 (1.67)	<.001	**<.001**
Child HSC (AES)	5.56 (1.02)	5.28 (1.17)	<.001	**<.001**	5.27 (1.19)	5.15 (1.18)	.067	.10
Behavioural control	14.39 (1.51)	13.52 (2.38)	<.001	**<.001**	14.48 (1.35)	13.62 (2.50)	<.001	**<.001**
Positive home experiences (PHE)	25.15 (4.60)	24.12 (4.58)	<.001	**<.001**	25.07 (4.82)	24.64 (4.12)	.017	**.029**
Physical abuse (mother's report)	8.47 (6.77)	9.68 (6.83)	<.001	**<.001**	6.74 (6.25)	7.41 (6.89)	.28	.33
Perceived refugee environment	3.16 (0.51)	3.27 (0.50)	<.001	**<.001**	3.32 (0.46)	3.32 (0.53)	.68	.68
Human insecurity	3.71 (0.39)	3.66 (0.40)	.003	**.004**	3.72 (0.38)	3.76 (0.34)	.18	.25
Bullying	3.24 (4.60)	4.74 (5.55)	<.001	**<.001**	3.15 (5.72)	4.93 (6.93)	<.001	**<.001**
Violent victimisation	0.48 (1.41)	0.82 (1.79)	<.001	**<.001**	0.27 (0.98)	0.56 (1.65)	.004	**.009**
Collective efficacy and informal social control	32.29 (6.55)	31.40 (6.46)	.004	**.004**	31.37 (8.36)	31.76 (7.49)	.56	.61

Bold values are statistically significant at the 5% level (*p* < .05).

^a^ Mean (*SD*).

^b^ Wilcoxon rank sum test.

^c^ False discovery rate correction for multiple testing.

We first conducted a global test of no regression by including all candidate predictors in a single model (Table S6). Because the fit of this model was significant (*F*[1,341, 67] = 4.74, *p* < .0001), refined selection of individual predictors was supported (Harrell, 2015).

**Figure 2:**
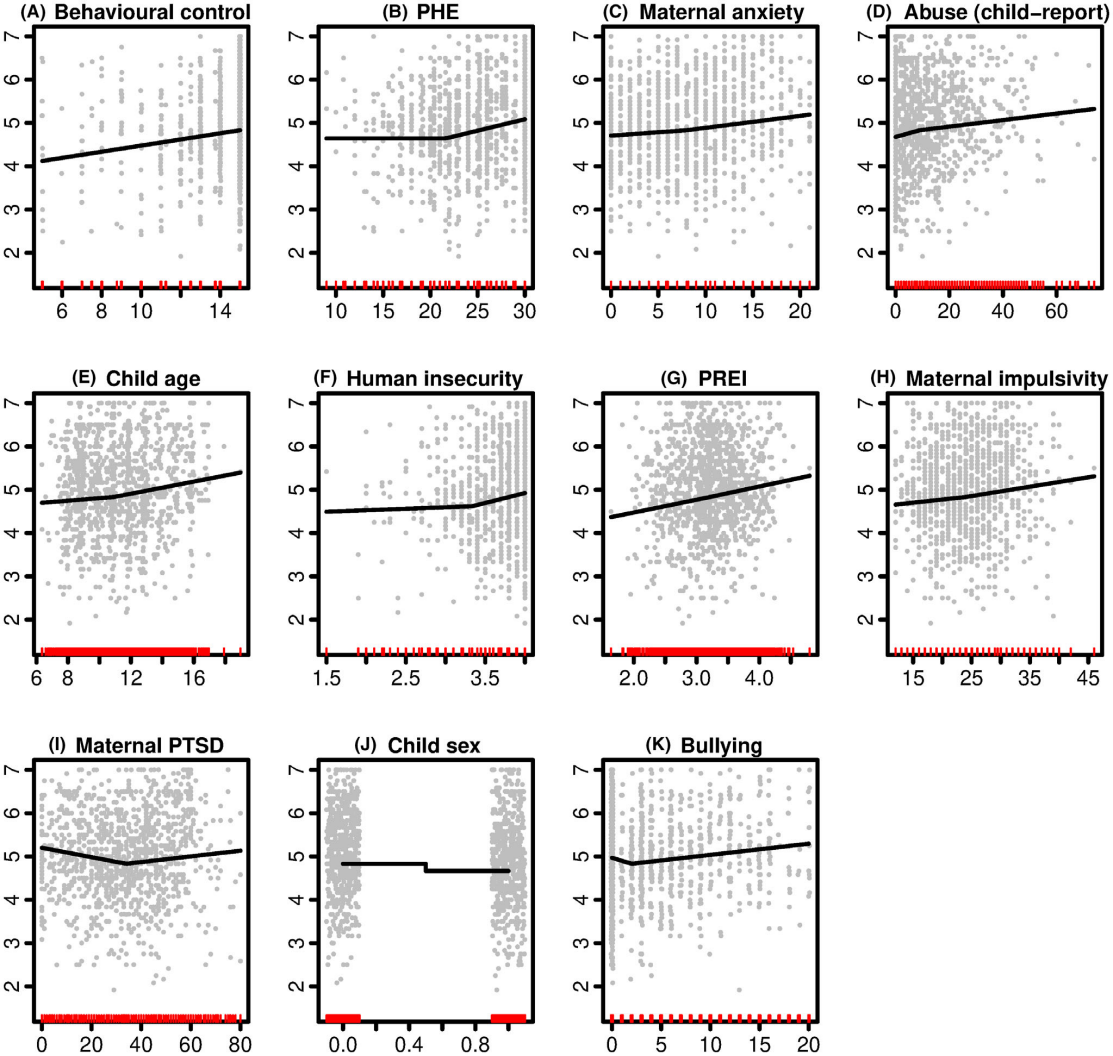
Regression line fits of selected predictors for total environmental sensitivity. Predictors are ordered according to model fit importance from a to k. In all graphs, average item score for the HSC scale is represented by the *y*‐axis, while the *x*‐axis indicates average item or total scale scores for each predictor (controlling for all other predictors in the model). Child sex was coded ‘0’ for females and ‘1’ for males. Grey points indicate raw data, while red marks indicate *x*‐axis values at which raw data were observed. PHE, positive home experiences; PREI, perceived refugee environment index; PTSD, post‐traumatic stress disorder

We began predictor selection in the training data set using MARS, first treating sensitivity as a single scale. Of the more than 40 possible predictors, 11 were selected. Predictors and their coefficients are listed in Table 3, in order of estimated importance (defined by the number of model subsets in which they remained during backward selection, and their relative effect in improving fit statistics scaled against the best predictor, which is set at a value of 100). Regression line fit per predictor is shown in Figure 2. Interestingly, some variables evinced non‐linear relationships to sensitivity that were better described by two piecewise regression splines (or hinges).

**Table 3:** MARS model fit for sensitivity as a single scale

Variables	Number of model subsets	GCV	RSS	Coefficient	Estimate
Behavioural control	19	100.00	100.00		0.07
Positive home experiences	18	80.68	86.767	h(PHE‐21.6)	0.05
Maternal anxiety	17	69.25	78.85	h(Anxiety‐8)	0.03
				h(8‐Anxiety)	−0.02
Child‐reported abuse (total)	16	55.55	70.14	h(Abuse‐9)	0.01
				h(9‐Abuse)	−0.02
Child age	15	43.84	63.09	h(Age‐10.92)	0.07
				h(10.92‐Age)	−0.03
Human insecurity	14	31.43	56.51	h(Insecurity‐3.33)	0.46
				h(3.33‐Insecurity)	−0.07
PREI	13	17.60	50.74	h(PREI‐3.21)	0.31
				h(3.21‐PREI)	−0.30
Maternal impulsivity	12	−13.89	45.49	h(Impulsivity‐24)	0.02
				h(24‐Impulsivity)	−0.02
Maternal PTSD	11	−21.16	41.53	h(PTSD‐34)	0.01
				h(34‐PTSD)	0.01
Sex (male)	10	−25.68	37.52		−0.17
Bullying	9	−28.18	33.78	h(Bullying‐2)	0.03
				h(2‐Bullying)	0.07
Intercept					3.36

GCV, generalised cross validation; h, hinge; PREI, perceived refugee environment index; RSS, residual sum of squares.

**Figure 3:**
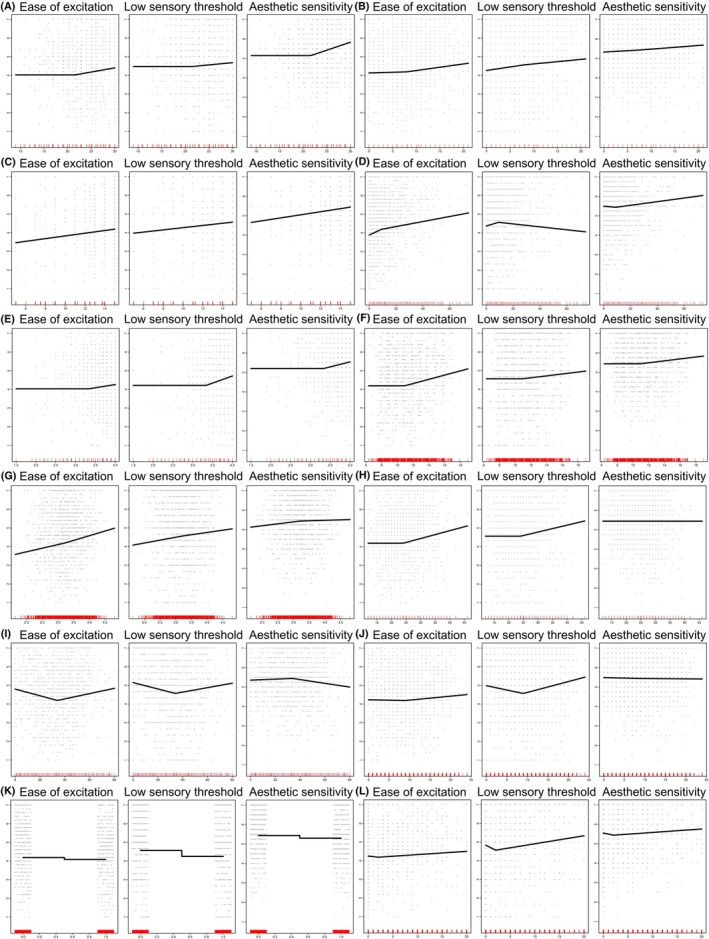
Regression line fits of selected predictors for environmental sensitivity subscales. Predictors are ordered according to model fit importance, namely: (A) positive home experiences; (B) maternal anxiety; (C) behavioural control; (D) child‐reported abuse; (E) human insecurity; (F) child age; (G) perceived refugee index; (H) maternal impulsivity; (I) maternal PTSD; (J) war exposure; (K) child sex and (L) bullying. For each predictor, three graphs are displayed for each of the subscales, namely Ease of Excitation (EOE), Low Sensory Threshold (LST) and Aesthetic Sensitivity (AES). In all graphs, average item score for the HSC scale is represented by the *y*‐axis, while the *x*‐axis indicates average item or total scale scores for each predictor. Child sex was coded ‘0’ for females and ‘1’ for males. Grey points indicate raw data, while red marks indicate *x*‐axis values at which raw data were observed

For sensitivity subscales, three models were simultaneously built and optimised. Twelve predictors were selected, including the above 11 predictors, as well as war exposure. Regression coefficients are displayed in Table 4, and line fits are shown in Figure 3. War exposure predicted increases in EOE and LST, but shared no meaningful relationship with AES. The estimated importance of predictor variables changed marginally, but paralleled results for ES as a single scale. Model diagnostics did not reveal issues with heteroscedasticity; however, QQ plots suggested some deviation in residuals (Figure S1). In follow‐up analyses, eight of the 12 predictors were also selected across stepwise (Table S12), LASSO, and elastic net regression techniques (Table S13). Child sex, bullying and war exposure were not consistently selected across these methods, while maternal PTSD was never selected. We then compared the fit of the MARS models between training and test data. Performance in the test data declined, with larger error terms and approximately 5% less of the variance in sensitivity explained (Table 5).

**Table 4:** MARS model fit for sensitivity subscales

Variables	Number of model subsets	GCV	RSS	Coefficient	EOE	LST	AES
Positive home experiences	18	100.00	100.00	h(PHE‐21.6)	0.05	0.02	0.08
Maternal anxiety	17	81.25	89.56	h(Anxiety‐8)	0.04	0.02	0.02
				h(8‐Anxiety)	−0.01	−0.04	−0.01
Behavioural control	16	67.98	82.16		0.07	0.06	0.08
Abuse (total)	15	53.47	74.80	h(Abuse‐9)	0.01	0.01	0.01
				h(9‐Abuse)	−0.03	−0.02	0.01
Human insecurity	14	41.21	68.84	h(Insecurity‐3.33)	0.34	0.76	0.51
Child age	13	27.58	63.22	h(Age‐10.92)	0.11	0.05	0.05
PREI	12	9.05	58.18	h(PREI‐3.21)	0.50	0.24	0.05
				h(3.21‐PREI)	−0.39	−0.31	−0.20
Maternal impulsivity	11	−11.65	54.78	h(Impulsivity‐24)	0.04	0.04	0.00
Maternal PTSD	10	−19.83	50.92	h(PTSD‐34)	0.01	0.01	0.01
				h(34‐PTSD)	0.02	0.02	0.00
War exposure	9	−22.90	47.44	h(War Exposure‐9)	0.02	0.06	−0.00
				h(9‐War Exposure)	0.00	0.05	0.00
Sex (male)	8	−28.18	42.92		−0.10	−0.32	−0.14
Bullying	5	−30.87	30.57	h(Bullying‐2)	0.02	0.04	0.02
				h(2‐Bullying)	0.03	0.14	0.06
Intercept					2.78	3.24	3.73

GCV, generalised cross validation; h, hinge; PREI, perceived refugee environment index; RSS, residual sum of squares.

**Table 5:** Model fits

Set	RMSE	*R* ^2^	MAE
Total scale			
Train	0.917	.169	0.736
Test	0.982	.119	0.779
Subscales			
Train	1.302	.153	1.037
Test	1.354	.115	1.066

MAE, mean absolute error; RMSE, root mean square error.

Additionally, we grouped children into three age categories and refitted the model. Sensitivity in children (younger than 10) was commonly informed by maternal acceptance, maternal anxiety, positive home experiences, and self‐reported physical abuse (Table S7). For pre‐teens (10–12 years old), only behavioural control and human insecurity predicted sensitivity (Table S8). Finally, for teenagers (13 and older), maternal anxiety, positive home experiences, mental health, and age were commonly selected predictors of sensitivity (Table S9). When stratified by sex, selected variables in boys included positive home experiences, behavioural control, verbal abuse, and human insecurity (Table S10), while for girls, maternal anxiety, child age, refugee environment, and war exposure were most frequently selected (Table S11).

**Figure 4:**
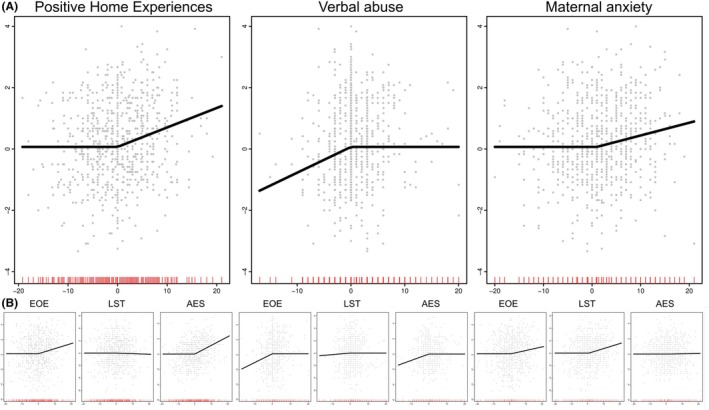
Regression line fits of selected predictors for environmental sensitivity changes from Wave 1 to 2. Regression line fits are shown for (A) total scores on the HSC and (B) subscales per each of the predictors in panel A. In all graphs, change in average item score for the HSC scale is represented by the *y*‐axis, while the *x*‐axis indicates changes in total scale scores for each predictor. Grey points indicate raw data, while red marks indicate *x*‐axis values at which raw data were observed

Finally, we explored changes in environmental sensitivity from Wave 1 to Wave 2. Only three predictors were selected for the model. Decreasing child‐reported verbal abuse was associated with declining sensitivity levels, while increasing positive home experiences and maternal anxiety were linked to rising levels of sensitivity (Figure 4). Regarding sensitivity subscales, positive home experiences had no impact on changes in LST, and maternal anxiety did not affect changes in AES. As an alternative approach (Table S14), we also regressed sensitivity at Wave 2 onto Wave 2 scores, while controlling for Wave 1 scores. This approach similarly highlighted the importance of verbal abuse and positive home experiences at Wave 2 but did not suggest a strong role for maternal anxiety.

## Discussion

Individuals differ in their sensitivity to environmental influences, including the impact of war exposure and forced displacement. Such variability is shaped by genetic and environmental factors (Assary et al., 2021), but few predictors of sensitivity have been identified. Here, we examined potential individual‐, family‐ and community‐level predictors of ES in Syrian refugee children based in Lebanon. Twelve variables emerged as possible linear and non‐linear predictors of ES (and its subscales), the five most frequently selected of which were behavioural control, positive home experiences, maternal anxiety, human insecurity, and child‐reported child abuse. Although we hypothesised that only adversity‐related variables would emerge as predictors in this harsh context, supportive factors were also associated. However, effect estimates for all predictors were small, and their relative importance differed among sexes and age groups.

### Predictors of sensitivity

Previous research has documented increases in children's sensitivity with increasing environmental adversity, such as prenatal maternal stress (May et al., 2023). Likewise, we identified heightened sensitivity in children reporting more abusive rearing practices and belonging to anxious mothers reporting high human insecurity. Children experiencing abuse typically develop personalities that are subdued, shy, and withdrawn (Oates, 1984), characterised by higher levels of neuroticism and openness (Allen & Lauterbach, 2007; Lee & Song, 2017). Relatedly, highly sensitive persons are often considered shy, frequently withdraw into solitude (Aron & Aron, 1997), and tend to report greater neuroticism (in childhood and adulthood) and openness (in adulthood; Lionetti et al., 2019; Pluess et al., 2023). Across measurement waves, sensitivity levels appeared to decline if children reported reductions in verbal abuse, further supporting a link between abuse and sensitivity. Studies suggest that the risk of child abuse rises when mothers suffer from mental health issues, including anxiety (Douki et al., 2013; Jakupčević & Ajduković, 2011), as well as within households enduring economic insecurity (a form of human insecurity; Conrad‐Hiebner & Byram, 2020), possibly explaining the co‐emergence of these predictors in our study.

Higher childhood sensitivity has also been noted following exposure to supportive contexts, including high perceived early‐life maternal care (Engert et al., 2010), low psychosocial stress (Seery, Holman, & Silver, 2010) and non‐restrictive parenting (Shakiba, Ellis, Bush, & Boyce, 2020). Here, positive home experiences (how frequently children noted the presence of their parents during meal‐, play‐ and bedtime, and whether children considered their home a fun and safe environment) were estimated as important, particularly at the highest levels of measurement. Positive home experiences also predicted increases in sensitivity (particularly EOE and AES) across waves. In addition, parental behavioural control, a measure of the efforts parents dedicate to monitor their child's adherence to consistent expectations of behaviour (Gittins & Hunt, 2019), was linearly associated with increasing sensitivity. Behavioural control is considered beneficial for young children, providing them clear guidelines regarding actions and their associated consequences. Coincidentally, stronger behavioural control tends to be exerted by parents of Arabic and Eastern collectivistic cultures where interdependent relationships are emphasised (Wong, Zhuang, & Ng, 2019) and sensitive dispositions are valued (Aron, 2004), and potentially more prevalent (Gureyev et al., 2016; Homberg, Schubert, Asan, & Aron, 2016). Behavioural control correlated significantly with positive home experiences (*r* = 0.29, *p* < .001, Table S4), explaining the emergence of both of these predictors. However, behavioural control has been implicated in greater anxiety (McLeod, Wood, & Weisz, 2007), harsher self‐criticism, and lower self‐esteem and self‐efficacy (Wong et al., 2019), especially among adolescents (Gittins & Hunt, 2019). These issues are frequently reported by highly sensitive persons (Black & Kern, 2020), suggesting that while firm control is beneficial and may calibrate children towards greater sensitivity, this possibly renders them more susceptible to internalising problems.

Some variables had V‐shape relationships to sensitivity, including maternal PTSD, bullying, and war exposure. In these instances, sensitivity appeared heightened at both ends of the scoring spectrum but declined at intermediate levels. Considered together with other selected predictor variables, our findings suggest that both highly supportive *and* highly adverse environments associate with greater childhood sensitivity. Meanwhile, children in more moderate environments reported less sensitivity. Such findings align with ES theorising (Pluess & Belsky, 2011), the U‐shaped development of stress reactivity (Boyce & Ellis, 2005) and those of Li et al. (2021) described earlier. Maternal PTSD evinced the clearest V‐shaped relationship with sensitivity (Figure 2), but other selection techniques (stepwise, LASSO, and elastic net regression) did not consider PTSD an informative predictor. Consequently, strictly linear models may not be suitable for identifying all sensitivity predictors.

Of the emerging predictors of sensitivity, many encapsulated significant sex differences. Aside from girls tending to report greater levels of sensitivity (observed elsewhere, e.g. May, Norris, Richter, & Pitman, 2020), they also noted more positive home experiences, fewer instances of bullying, and greater behavioural control exerted by their mothers compared to boys. Correspondingly, mothers of daughters (as opposed to sons) felt more insecure and had less favourable perceptions of their refugee environment. When stratifying the sample by sex, predictors of sensitivity in girls were mainly maternal anxiety, war exposure, and the perceived refugee environment, while for boys, positive home experiences, behavioural control, verbal abuse, and human insecurity were commonly selected. Together, these observations highlight how sensitivity may be differentially influenced in boys and girls – an issue magnified in our sample by both cultural and refugee‐related concerns. Syrian children are reared with different gender roles and expectations, based on traditional Islamic cultural values (Almalki, 2020), and also face different pressures in the refugee environment. While boys are often subjected to bullying and child labour, girls are entered into arranged marriages, are at risk of sexual exploitation and assault, and have highly restricted mobility (DeJong et al., 2017; Sirin & Rogers‐Sirin, 2015).

Child age also appeared to alter which predictors were more salient to sensitivity levels. The sensitivity of children tended to associate with maternal acceptance, maternal anxiety, positive home experiences, and self‐reported abuse. For pre‐teens, only behavioural control and human insecurity tracked with sensitivity, while for teens, maternal anxiety, positive home experiences and self‐reported mental health aligned with sensitivity levels. The changing importance of predictors may reflect the shifting value of contextual influences as children mature, but might also be symptomatic of power and variance differences across subsamples.

Against previous evidence (Weyn et al., 2025), polygenic scores for ASD and ADHD were negligible predictors of sensitivity. ASD and ADHD are conceptually different phenotypes from high sensitivity, with measurable differences in brain activation (Acevedo et al., 2018) and thus polygenic scores for these phenotypes may be weak predictors. Prediction may have been further weakened due to the deflation in power when applying European‐ancestry derived scores to Middle Eastern individuals (Privé et al., 2022). Moreover, the utility of polygenic scores in psychosocial research remains questionable, given many confounding issues (Burt, 2022). Future research is needed to unpack genetic variants conferring interindividual differences in sensitivity and related traits.

### Strengths and limitations

There are several key strengths to this study. Firstly, our sample was large and comprehensively surveyed, providing numerous psychosocial and environmental variables across two time points, along with sufficient power to conduct rigorous statistical testing. Secondly, genotypic data allowed us to investigate ES predictors at an additional level of analysis. Lastly, the non‐European ethnicity of refugee participants and their location within a low‐and‐middle‐income host country helped to address biases in the current literature (Fatumo et al., 2022; Stevens, Siraj, & Kong, 2023).

A number of limitations should also be noted. Statistical modelling at the tail ends of a continuous variable tends to have reduced accuracy for several reasons (e.g. lower sample size, zero‐inflated responses), and thus the slope of our regression splines might be overly influenced, resulting in artificial V‐shapes, although no serious heteroscedasticity was detected in any of the models. Furthermore, during iterative model fitting, some variables were prone to omission in the selection process, possibly highlighting the acknowledged arbitrariness of MARS. Relatedly, due to the design of our study, causality cannot be inferred between identified predictors and sensitivity. This is a considerable issue because many of the observable behavioural responses engendered by a low‐threshold nervous system can be mistakenly attributed to other causes (Aron, 2006). Whether children evince inhibited behaviour due to a biologically embedded lower tolerance for sensory stimulation, or out of fear learned through operant conditioning, are two fundamentally different scenarios distinguished only through covert cognitive processes not reliably captured by behavioural observation or self‐report instrumentation (Aron & Aron, 1997). In our study, refugee children may have self‐reported greater sensitivity due to a post‐traumatic hypersensitivity response, potentially inflating associations with abuse and war exposure, but it remains uncertain whether these effects drive mechanistic changes in sensitivity or only condition comparable behavioural responses nevertheless captured by self‐report instrumentation. A final limitation is the restricted generalisability of our findings, possibly highlighted by the weaker fit of our regression models when applied to test data. However, our sample was drawn from a niche context, and if the calibration of sensitivity is influenced by an assortment of genetic and environmental forces (Assary et al., 2021), then it will remain difficult to identify generalisable signals against the background of such variability. Future research should seek to replicate our results in independent and contextually diverse samples, while also considering additional markers of sensitivity.

### Conclusion

In conclusion, our study further supports theoretical claims that ES is heightened in both adverse *and* supportive contexts. Predictor variables relating to caregiver mental health (e.g. anxiety, PTSD, human insecurity), parenting quality (e.g. abuse, behavioural control, positive home experiences) and environmental harshness (e.g. bullying, war exposure) may serve as useful estimators of children's emerging sensitivity levels. Gauging these differing sensitivity levels might be beneficial in the refugee context, helping to identify children at risk of mental health problems, but also disproportionately receptive to positive intervention efforts.

## Ethical considerations

All adult participants (18 years or older) provided written informed consent to participate in this study. Children (younger than 18 years old) provided verbal assent. Ethical approval for the study was obtained from the Institutional Review Board at the University of Balamand/Saint George Hospital University Medical Center, Lebanon (ref: IRB/O/024‐16/1815), the Lebanese National Consultative Committee on Ethics and the Ministry of Public Health in Lebanon.

**Key points**

**What's known**

- Refugee children differ in their response to adversity, partly due to differences in Environmental Sensitivity.
- Environmental Sensitivity is shaped by both environmental and genetic factors, but few predictor variables have been elucidated.

**What's new**

- In a large sample of Syrian refugee children, we identified maternal anxiety, human insecurity, behavioural control, positive home experiences, and child‐reported child abuse as robust predictors of Environmental Sensitivity.
- Child‐reported verbal abuse, maternal anxiety, and positive home experiences also predicted changes in sensitivity over a 1‐year period.

**What's relevant**

- Knowledge of Environmental Sensitivity predictors may help to stratify refugee children by sensitivity, improving the clinical care and treatment offered to them.

## Supporting information


**Table S1.** Polygenic scores used.
**Table S2.** Descriptive statistics for study participants by sex.
**Table S3.** Peason correlations between individual‐level variables.
**Table S4.** Pearson correlations between family‐level variables.
**Table S5.** Pearson correlations between community‐level variables.
**Table S6.** Global test of no regression.
**Table S7.** Predictor importance for children younger than 10.
**Table S8.** Predictor importance for children between 10 and 12 years old.
**Table S9.** Predictor importance for children aged 13 and older.
**Table S10.** Predictor importance for boys.
**Table S11.** Predictor importance for girls.
**Table S12.** Variables selected via stepwise selection.
**Table S13.** Variables selected via penalised regression.
**Table S14.** Predictors of residual difference in sensitivity from Wave 1 to Wave 2.
**Figure S1.** MARS model diagnostics.

## Data Availability

Data are available upon request to the corresponding author.
