# Supplementary material for: Predictors of Environmental Sensitivity in Syrian refugee children
Source: J Child Psychol Psychiatry. 2025 Jun 10;66(11):1688–702. doi: 10.1111/jcpp.14178 (PMC12354167; doi:10.1111/jcpp.14178)
Supplement: Supplementary file 1 — Table S1. Polygenic scores used. Table S2. Descriptive statistics for study participants by sex. Table S3. Peason correlations between individual‐level variables. Table S4. Pearson correlations between family‐level variables. Table S5. Pearson correlations between community‐level variables. Table S6. Global test of no regression. Table S7. Predictor importance for children younger than 10. Table S8. Predictor importance for children between 10 and 12 years old. Table S9. Predictor importance for children aged 13 and older. Table S10. Predictor importance for boys. Table S11. Predictor importance for girls. Table S12. Variables selected via stepwise selection. Table S13. Variables selected via penalised regression. Table S14. Predictors of residual difference in sensitivity from Wave 1 to Wave 2. Figure S1. MARS model diagnostics. [file JCPP-66-1688-s001.docx]

# Supplementary information

| Table S1: Polygenic scores used   \| Polygenic Score ID & Name \| PGS Publication ID (PGP) \| Reported Trait \| Number of Variants \| Coverage (%) in study sample \| \| --- \| --- \| --- \| --- \| --- \| \| PGS002746 (PRS_ADHD) \| PGP000358 \|Lahey BB et al. J Psychiatr Res (2022) \| Attention-deficit hyperactivity disorder \| 513,659 \| 91.77 \| \| PGS003753 (PRS35445_ADHD) \| PGP000473 \|Sato JR et al. Genes Brain Behav (2023) \| Attention deficit hyperactivity disorder \| 35,445 \| 100.00 \| \| PGS000327 (ASD2019) \| PGP000098 \|Grove J et al. Nat Genet (2019) \| Autism spectrum disorder \| 35,087 \| 93.59 \| \| PGS002790 (ASD_SDPR) \| PGP000370 \|Gui Y et al. Transl Psychiatry (2022) \| Autism spectrum disorder \| 916,713 \| 80.64 \| |
| --- | --- | --- | --- | --- | --- | --- | --- | --- | --- | --- | --- | --- | --- | --- | --- | --- | --- | --- | --- | --- | --- | --- | --- | --- | --- |

| Table S2: Descriptive statistics for study participants by sex   \|  \| **Wave 1** \| \| \| \| **Wave 2** \| \| \| \| \| --- \| --- \| --- \| --- \| --- \| --- \| --- \| --- \| --- \| \| **Variable** \| **Females** N = 735^1^ \| **Males** N = 674^1^ \| **p-value**^2^ \| **q-value**^3^ \| **Females** N = 735^1^ \| **Males** N = 674^1^ \| **p-value**^2^ \| **q-value**^3^ \| \| Child Age (years) \| 11.22 (2.41) \| 11.38 (2.35) \| 0.13 \| 0.20 \| 12.18 (2.40) \| 12.01 (2.24) \| 0.43 \| 0.65 \| \| Child HSC (total) \| 5.16 (0.98) \| 4.89 (1.03) \| <0.001 \| **<0.001** \| 4.94 (0.94) \| 4.61 (0.94) \| <0.001 \| **<0.001** \| \| Child HSC (EOE) \| 4.81 (1.40) \| 4.64 (1.40) \| 0.017 \| 0.052 \| 4.54 (1.41) \| 4.29 (1.45) \| 0.015 \| 0.053 \| \| Child HSC (LST) \| 5.19 (1.52) \| 4.78 (1.70) \| <0.001 \| **<0.001** \| 5.18 (1.57) \| 4.45 (1.67) \| <0.001 \| **<0.001** \| \| Child HSC (AES) \| 5.56 (1.02) \| 5.28 (1.17) \| <0.001 \| **<0.001** \| 5.27 (1.19) \| 5.15 (1.18) \| 0.067 \| 0.14 \| \| PGS000327 \| 10.85 (5.27) \| 10.80 (5.19) \| 0.95 \| 0.98 \| 10.85 (5.27) \| 10.80 (5.19) \| 0.95 \| 0.95 \| \| PGS002790 \| 0.17 (0.09) \| 0.18 (0.08) \| 0.94 \| 0.98 \| 0.17 (0.09) \| 0.18 (0.08) \| 0.94 \| 0.95 \| \| PGS002746 \| 225.17 (165.14) \| 229.29 (173.85) \| 0.79 \| 0.95 \| 225.17 (165.14) \| 229.29 (173.85) \| 0.79 \| 0.95 \| \| PGS003753 \| 8.88 (5.58) \| 9.51 (5.76) \| 0.035 \| 0.090 \| 8.88 (5.58) \| 9.51 (5.76) \| 0.035 \| 0.10 \| \| Mother age (years) \| 38.22 (7.27) \| 38.82 (7.49) \| 0.15 \| 0.23 \| 39.24 (7.41) \| 39.35 (7.86) \| 0.89 \| 0.95 \| \| Highest level of mother’s education \|  \|  \| 0.91 \| 0.98 \|  \|  \| 0.72 \| 0.95 \| \| *Did not attend school* \| 428.00 (58.55%) \| 384.00 (56.97%) \|  \|  \| 274.00 (58.17%) \| 235.00 (56.09%) \|  \|  \| \| *Up to Grade 12* \| 12.00 (1.64%) \| 10.00 (1.48%) \|  \|  \| 9.00 (1.91%) \| 5.00 (1.19%) \|  \|  \| \| *Up to Grade 6* \| 234.00 (32.01%) \| 228.00 (33.83%) \|  \|  \| 153.00 (32.48%) \| 145.00 (34.61%) \|  \|  \| \| *Up to Grade 9* \| 57.00 (7.80%) \| 52.00 (7.72%) \|  \|  \| 35.00 (7.43%) \| 34.00 (8.11%) \|  \|  \| \| Number of children \| 6.03 (2.39) \| 5.90 (2.48) \| 0.23 \| 0.31 \| 6.03 (2.39) \| 5.90 (2.48) \| 0.23 \| 0.39 \| \| Birth order of participating child \| 3.23 (2.48) \| 3.46 (2.52) \| 0.050 \| 0.10 \| 3.23 (2.48) \| 3.46 (2.52) \| 0.050 \| 0.12 \| \| Maternal HSP (total) \| 5.24 (0.87) \| 5.34 (0.90) \| 0.021 \| 0.058 \| 5.18 (0.86) \| 5.23 (0.88) \| 0.27 \| 0.44 \| \| Maternal PTSD \| 33.48 (17.82) \| 33.79 (17.86) \| 0.75 \| 0.93 \| 24.58 (18.68) \| 24.05 (17.68) \| 0.85 \| 0.95 \| \| Maternal anxiety \| 8.16 (5.11) \| 8.29 (5.58) \| 0.85 \| 0.98 \| 6.54 (5.32) \| 7.24 (5.32) \| 0.039 \| 0.10 \| \| Maternal depression \| 15.21 (6.16) \| 15.57 (6.72) \| 0.13 \| 0.20 \| 13.60 (7.75) \| 14.35 (6.95) \| 0.11 \| 0.20 \| \| Maternal impulsivity \| 24.17 (5.59) \| 24.32 (5.94) \| 0.93 \| 0.98 \| 21.67 (6.13) \| 22.88 (6.19) \| 0.002 \| **0.012** \| \| Mental health in the past 12 months (MH1) \| 4.34 (0.79) \| 4.30 (0.84) \| 0.65 \| 0.84 \| 3.90 (0.99) \| 4.11 (0.90) \| 0.002 \| **0.012** \| \| Mental health since onset of war, but prior to the past 12 months (MH2) \| 4.71 (0.58) \| 4.64 (0.67) \| 0.12 \| 0.20 \| 4.56 (0.75) \| 4.55 (0.76) \| 0.90 \| 0.95 \| \| Mental health prior to the onset of war (MH3) \| 2.07 (0.96) \| 2.17 (0.95) \| 0.044 \| 0.10 \| 1.85 (0.85) \| 1.88 (0.92) \| 0.85 \| 0.95 \| \| Total abuse (child-report) \| 11.44 (12.14) \| 12.23 (12.05) \| 0.088 \| 0.16 \| 7.80 (9.78) \| 9.13 (10.81) \| 0.064 \| 0.14 \| \| Psychological control \| 11.24 (2.62) \| 11.17 (2.51) \| 0.98 \| 0.98 \| 10.64 (2.12) \| 10.67 (2.29) \| 0.93 \| 0.95 \| \| Acceptance \| 27.47 (3.70) \| 27.05 (4.16) \| 0.060 \| 0.12 \| 27.72 (4.11) \| 27.32 (4.31) \| 0.072 \| 0.14 \| \| Behavioural control \| 14.39 (1.51) \| 13.52 (2.38) \| <0.001 \| **<0.001** \| 14.48 (1.35) \| 13.62 (2.50) \| <0.001 \| **<0.001** \| \| Mother-child conflict \| 6.01 (3.29) \| 6.02 (3.03) \| 0.29 \| 0.38 \| 7.31 (3.94) \| 7.24 (3.86) \| 0.84 \| 0.95 \| \| Positive Home Experiences (PHE) \| 25.15 (4.60) \| 24.12 (4.58) \| <0.001 \| **<0.001** \| 25.07 (4.82) \| 24.64 (4.12) \| 0.017 \| 0.054 \| \| Physical abuse (mother-report) \| 8.47 (6.77) \| 9.68 (6.83) \| <0.001 \| **0.002** \| 6.74 (6.25) \| 7.41 (6.89) \| 0.28 \| 0.44 \| \| Perceived refugee environment \| 3.16 (0.51) \| 3.27 (0.50) \| <0.001 \| **<0.001** \| 3.32 (0.46) \| 3.32 (0.53) \| 0.68 \| 0.94 \| \| Human insecurity \| 3.71 (0.39) \| 3.66 (0.40) \| 0.003 \| **0.013** \| 3.72 (0.38) \| 3.76 (0.34) \| 0.18 \| 0.33 \| \| Perceived Social Support \| 5.59 (0.94) \| 5.53 (0.93) \| 0.19 \| 0.27 \| 5.67 (0.99) \| 5.82 (0.99) \| 0.014 \| 0.053 \| \| Bullying \| 3.24 (4.60) \| 4.74 (5.55) \| <0.001 \| **<0.001** \| 3.15 (5.72) \| 4.93 (6.93) \| <0.001 \| **<0.001** \| \| Violent victimisation \| 0.48 (1.41) \| 0.82 (1.79) \| <0.001 \| **<0.001** \| 0.27 (0.98) \| 0.56 (1.65) \| 0.004 \| **0.018** \| \| Collective Efficacy and Informal Social Control \| 32.29 (6.55) \| 31.40 (6.46) \| 0.004 \| **0.013** \| 31.37 (8.36) \| 31.76 (7.49) \| 0.56 \| 0.80 \| \| War exposure \| 9.36 (5.41) \| 9.86 (5.59) \| 0.075 \| 0.14 \| 7.12 (5.67) \| 8.49 (6.39) \| 0.003 \| **0.015** \| \| Time since leaving Syria \|  \|  \| 0.038 \| 0.092 \|  \|  \| 0.038 \| 0.10 \| \| *1-2 years ago* \| 98.00 (13.35%) \| 103.00 (15.40%) \|  \|  \| 98.00 (13.35%) \| 103.00 (15.40%) \|  \|  \| \| *2-3 years ago* \| 98.00 (13.35%) \| 89.00 (13.30%) \|  \|  \| 98.00 (13.35%) \| 89.00 (13.30%) \|  \|  \| \| *3-4 years ago* \| 298.00 (40.60%) \| 229.00 (34.23%) \|  \|  \| 298.00 (40.60%) \| 229.00 (34.23%) \|  \|  \| \| *Less than 1 year ago* \| 139.00 (18.94%) \| 123.00 (18.39%) \|  \|  \| 139.00 (18.94%) \| 123.00 (18.39%) \|  \|  \| \| *More than 4 years ago* \| 101.00 (13.76%) \| 125.00 (18.68%) \|  \|  \| 101.00 (13.76%) \| 125.00 (18.68%) \|  \|  \| \| ^1^Mean (SD); n (%) \| \| \| \| \| \| \| \| \| \| ^2^Wilcoxon rank sum test; Pearson's Chi-squared test \| \| \| \| \| \| \| \| \| \| ^3^False discovery rate correction for multiple testing \| \| \| \| \| \| \| \| \| |
| --- | --- | --- | --- | --- | --- | --- | --- | --- | --- | --- | --- | --- | --- | --- | --- | --- | --- | --- | --- | --- | --- | --- | --- | --- | --- | --- | --- | --- | --- | --- | --- | --- | --- | --- | --- | --- | --- | --- | --- | --- | --- | --- | --- | --- | --- | --- | --- | --- | --- | --- | --- | --- | --- | --- | --- | --- | --- | --- | --- | --- | --- | --- | --- | --- | --- | --- | --- | --- | --- | --- | --- | --- | --- | --- | --- | --- | --- | --- | --- | --- | --- | --- | --- | --- | --- | --- | --- | --- | --- | --- | --- | --- | --- | --- | --- | --- | --- | --- | --- | --- | --- | --- | --- | --- | --- | --- | --- | --- | --- | --- | --- | --- | --- | --- | --- | --- | --- | --- | --- | --- | --- | --- | --- | --- | --- | --- | --- | --- | --- | --- | --- | --- | --- | --- | --- | --- | --- | --- | --- | --- | --- | --- | --- | --- | --- | --- | --- | --- | --- | --- | --- | --- | --- | --- | --- | --- | --- | --- | --- | --- | --- | --- | --- | --- | --- | --- | --- | --- | --- | --- | --- | --- | --- | --- | --- | --- | --- | --- | --- | --- | --- | --- | --- | --- | --- | --- | --- | --- | --- | --- | --- | --- | --- | --- | --- | --- | --- | --- | --- | --- | --- | --- | --- | --- | --- | --- | --- | --- | --- | --- | --- | --- | --- | --- | --- | --- | --- | --- | --- | --- | --- | --- | --- | --- | --- | --- | --- | --- | --- | --- | --- | --- | --- | --- | --- | --- | --- | --- | --- | --- | --- | --- | --- | --- | --- | --- | --- | --- | --- | --- | --- | --- | --- | --- | --- | --- | --- | --- | --- | --- | --- | --- | --- | --- | --- | --- | --- | --- | --- | --- | --- | --- | --- | --- | --- | --- | --- | --- | --- | --- | --- | --- | --- | --- | --- | --- | --- | --- | --- | --- | --- | --- | --- | --- | --- | --- | --- | --- | --- | --- | --- | --- | --- | --- | --- | --- | --- | --- | --- | --- | --- | --- | --- | --- | --- | --- | --- | --- | --- | --- | --- | --- | --- | --- | --- | --- | --- | --- | --- | --- | --- | --- | --- | --- | --- | --- | --- | --- | --- | --- | --- | --- | --- | --- | --- | --- | --- | --- | --- | --- | --- | --- | --- | --- | --- | --- | --- | --- | --- | --- | --- | --- | --- | --- | --- | --- | --- | --- | --- | --- | --- | --- | --- | --- | --- | --- | --- | --- | --- | --- | --- | --- | --- | --- | --- | --- | --- | --- | --- | --- | --- | --- | --- | --- | --- | --- | --- | --- | --- | --- | --- | --- | --- | --- | --- | --- | --- | --- | --- | --- | --- | --- | --- | --- | --- | --- | --- | --- | --- | --- | --- | --- | --- | --- | --- | --- | --- | --- | --- | --- | --- | --- | --- | --- | --- | --- | --- | --- | --- | --- | --- | --- | --- | --- | --- | --- | --- | --- | --- | --- |

| \| Table S3: Pearson correlations between individual-level variables   \|  \| HSC \| EOE \| LST \| AES \| PGS000327 \| PGS002790 \| PGS002746 \| PGS003753 \| \| --- \| --- \| --- \| --- \| --- \| --- \| --- \| --- \| --- \| \| HSC \|  \|  \|  \|  \|  \|  \|  \|  \| \| EOE \| 0.84*** \|  \|  \|  \|  \|  \|  \|  \| \| LST \| 0.73*** \| 0.41*** \|  \|  \|  \|  \|  \|  \| \| AES \| 0.62*** \| 0.28*** \| 0.25*** \|  \|  \|  \|  \|  \| \| PGS000327 \| 0.00 \| 0.02 \| 0.00 \| -0.02 \|  \|  \|  \|  \| \| PGS002790 \| 0.00 \| 0.01 \| 0.01 \| -0.03 \| 0.65*** \|  \|  \|  \| \| PGS002746 \| -0.03 \| -0.01 \| -0.04 \| -0.03 \| 0.12*** \| 0.23*** \|  \|  \| \| PGS003753 \| -0.05 \| -0.03 \| -0.04 \| -0.05 \| 0.18*** \| 0.25*** \| 0.54*** \|  \| \| \| --- \| --- \| --- \| --- \| --- \| --- \| --- \| --- \| --- \| --- \| --- \| --- \| --- \| --- \| --- \| --- \| --- \| --- \| --- \| --- \| --- \| --- \| --- \| --- \| --- \| --- \| --- \| --- \| --- \| --- \| --- \| --- \| --- \| --- \| --- \| --- \| --- \| --- \| --- \| --- \| --- \| --- \| --- \| --- \| --- \| --- \| --- \| --- \| --- \| --- \| --- \| --- \| --- \| --- \| --- \| --- \| --- \| --- \| --- \| --- \| --- \| --- \| --- \| --- \| --- \| --- \| --- \| --- \| --- \| --- \| --- \| --- \| --- \| --- \| --- \| --- \| --- \| --- \| --- \| --- \| --- \| --- \|   *p < .05; **p < .01; ***p < .001. HSC = Highly Sensitive Child scale; EOE = Ease of Excitation; LST = Low Sensory Threshold; AES = Aesthetic Sensitivity |
| --- | --- | --- | --- | --- | --- | --- | --- | --- | --- | --- | --- | --- | --- | --- | --- | --- | --- | --- | --- | --- | --- | --- | --- | --- | --- | --- | --- | --- | --- | --- | --- | --- | --- | --- | --- | --- | --- | --- | --- | --- | --- | --- | --- | --- | --- | --- | --- | --- | --- | --- | --- | --- | --- | --- | --- | --- | --- | --- | --- | --- | --- | --- | --- | --- | --- | --- | --- | --- | --- | --- | --- | --- | --- | --- | --- | --- | --- | --- | --- | --- | --- | --- |

| Table S4: Pearson correlations between family-level variables   \|  \| HSC \| EOE \| LST \| AES \| cICAST \| Psy. con. \| Accept \| Beh. con. \| Conflict \| PHE \| HSP \| pICAST \| HIS \| PTSD \| Anxiety \| Depr \| Impulsive \| MH1 \| MH2 \| MH3 \| Stress \| \| --- \| --- \| --- \| --- \| --- \| --- \| --- \| --- \| --- \| --- \| --- \| --- \| --- \| --- \| --- \| --- \| --- \| --- \| --- \| --- \| --- \| --- \| \| HSC \|  \|  \|  \|  \|  \|  \|  \|  \|  \|  \|  \|  \|  \|  \|  \|  \|  \|  \|  \|  \|  \| \| EOE \| 0.84*** \|  \|  \|  \|  \|  \|  \|  \|  \|  \|  \|  \|  \|  \|  \|  \|  \|  \|  \|  \|  \| \| LST \| 0.73*** \| 0.41*** \|  \|  \|  \|  \|  \|  \|  \|  \|  \|  \|  \|  \|  \|  \|  \|  \|  \|  \|  \| \| AES \| 0.62*** \| 0.28*** \| 0.25*** \|  \|  \|  \|  \|  \|  \|  \|  \|  \|  \|  \|  \|  \|  \|  \|  \|  \|  \| \| cICAST \| 0.08** \| 0.13*** \| -0.01 \| 0.03 \|  \|  \|  \|  \|  \|  \|  \|  \|  \|  \|  \|  \|  \|  \|  \|  \|  \| \| Psy. con. \| 0.05 \| 0.06* \| 0.00 \| 0.05 \| 0.34*** \|  \|  \|  \|  \|  \|  \|  \|  \|  \|  \|  \|  \|  \|  \|  \|  \| \| Accept \| 0.10*** \| 0.03 \| 0.06* \| 0.16*** \| -0.20*** \| -0.36*** \|  \|  \|  \|  \|  \|  \|  \|  \|  \|  \|  \|  \|  \|  \|  \| \| Beh. con. \| 0.20*** \| 0.13*** \| 0.12*** \| 0.23*** \| -0.09*** \| -0.08** \| 0.20*** \|  \|  \|  \|  \|  \|  \|  \|  \|  \|  \|  \|  \|  \|  \| \| Conflict \| 0.06* \| 0.08** \| 0.02 \| 0.01 \| 0.33*** \| 0.30*** \| -0.17*** \| -0.06* \|  \|  \|  \|  \|  \|  \|  \|  \|  \|  \|  \|  \|  \| \| PHE \| 0.18*** \| 0.08** \| 0.08** \| 0.28*** \| -0.15*** \| -0.09** \| 0.26*** \| 0.29*** \| -0.12*** \|  \|  \|  \|  \|  \|  \|  \|  \|  \|  \|  \|  \| \| HSP \| 0.09** \| 0.05 \| 0.07** \| 0.08** \| 0.04 \| 0.02 \| 0.00 \| 0.04 \| 0.11*** \| 0.11*** \|  \|  \|  \|  \|  \|  \|  \|  \|  \|  \|  \| \| pICAST \| 0.00 \| 0.05 \| -0.04 \| -0.03 \| 0.19*** \| 0.12*** \| -0.10*** \| -0.03 \| 0.02 \| -0.01 \| 0.01 \|  \|  \|  \|  \|  \|  \|  \|  \|  \|  \| \| HIS \| 0.14*** \| 0.08** \| 0.15*** \| 0.10*** \| -0.01 \| -0.03 \| 0.02 \| 0.10*** \| 0.05 \| 0.07** \| 0.19*** \| -0.10*** \|  \|  \|  \|  \|  \|  \|  \|  \|  \| \| PTSD \| 0.08** \| 0.07** \| 0.06* \| 0.03 \| 0.04 \| 0.08** \| -0.13*** \| -0.01 \| 0.01 \| 0.11*** \| 0.31*** \| 0.18*** \| 0.18*** \|  \|  \|  \|  \|  \|  \|  \|  \| \| Anxiety \| 0.15*** \| 0.12*** \| 0.14*** \| 0.07** \| 0.02 \| 0.01 \| -0.06* \| 0.02 \| 0.02 \| 0.03 \| 0.23*** \| 0.15*** \| 0.30*** \| 0.58*** \|  \|  \|  \|  \|  \|  \|  \| \| Depr \| 0.07** \| 0.06* \| 0.09*** \| 0.01 \| 0.14*** \| 0.06* \| -0.07* \| 0.01 \| 0.04 \| 0.03 \| 0.26*** \| 0.16*** \| 0.33*** \| 0.56*** \| 0.64*** \|  \|  \|  \|  \|  \|  \| \| Impulsive \| 0.09** \| 0.10*** \| 0.07* \| 0.01 \| 0.02 \| 0.07** \| -0.11*** \| 0.03 \| -0.03 \| 0.00 \| -0.09** \| 0.18*** \| 0.08** \| 0.18*** \| 0.28*** \| 0.33*** \|  \|  \|  \|  \|  \| \| MH1 \| 0.07** \| 0.03 \| 0.08** \| 0.05* \| 0.13*** \| 0.06* \| -0.04 \| 0.01 \| 0.06* \| 0.04 \| 0.20*** \| 0.06* \| 0.31*** \| 0.36*** \| 0.39*** \| 0.58*** \| 0.23*** \|  \|  \|  \|  \| \| MH2 \| 0.09*** \| 0.07** \| 0.07** \| 0.07* \| 0.02 \| 0.03 \| -0.01 \| 0.01 \| 0.09*** \| 0.06* \| 0.17*** \| -0.01 \| 0.16*** \| 0.12*** \| 0.17*** \| 0.20*** \| -0.03 \| 0.30*** \|  \|  \|  \| \| MH3 \| 0.08** \| 0.05* \| -0.01 \| 0.14*** \| 0.04 \| 0.07** \| -0.07* \| 0.01 \| 0.03 \| 0.09*** \| -0.01 \| 0.05 \| 0.01 \| 0.08** \| 0.03 \| 0.07* \| 0.16*** \| 0.17*** \| 0.06* \|  \|  \| \| Stress \| 0.04 \| 0.05 \| 0.04 \| -0.01 \| 0.10*** \| 0.00 \| -0.03 \| 0.01 \| 0.02 \| 0.02 \| 0.20*** \| 0.16*** \| 0.23*** \| 0.41*** \| 0.45*** \| 0.63*** \| 0.23*** \| 0.42*** \| 0.15*** \| 0.01 \|  \| |
| --- | --- | --- | --- | --- | --- | --- | --- | --- | --- | --- | --- | --- | --- | --- | --- | --- | --- | --- | --- | --- | --- | --- | --- | --- | --- | --- | --- | --- | --- | --- | --- | --- | --- | --- | --- | --- | --- | --- | --- | --- | --- | --- | --- | --- | --- | --- | --- | --- | --- | --- | --- | --- | --- | --- | --- | --- | --- | --- | --- | --- | --- | --- | --- | --- | --- | --- | --- | --- | --- | --- | --- | --- | --- | --- | --- | --- | --- | --- | --- | --- | --- | --- | --- | --- | --- | --- | --- | --- | --- | --- | --- | --- | --- | --- | --- | --- | --- | --- | --- | --- | --- | --- | --- | --- | --- | --- | --- | --- | --- | --- | --- | --- | --- | --- | --- | --- | --- | --- | --- | --- | --- | --- | --- | --- | --- | --- | --- | --- | --- | --- | --- | --- | --- | --- | --- | --- | --- | --- | --- | --- | --- | --- | --- | --- | --- | --- | --- | --- | --- | --- | --- | --- | --- | --- | --- | --- | --- | --- | --- | --- | --- | --- | --- | --- | --- | --- | --- | --- | --- | --- | --- | --- | --- | --- | --- | --- | --- | --- | --- | --- | --- | --- | --- | --- | --- | --- | --- | --- | --- | --- | --- | --- | --- | --- | --- | --- | --- | --- | --- | --- | --- | --- | --- | --- | --- | --- | --- | --- | --- | --- | --- | --- | --- | --- | --- | --- | --- | --- | --- | --- | --- | --- | --- | --- | --- | --- | --- | --- | --- | --- | --- | --- | --- | --- | --- | --- | --- | --- | --- | --- | --- | --- | --- | --- | --- | --- | --- | --- | --- | --- | --- | --- | --- | --- | --- | --- | --- | --- | --- | --- | --- | --- | --- | --- | --- | --- | --- | --- | --- | --- | --- | --- | --- | --- | --- | --- | --- | --- | --- | --- | --- | --- | --- | --- | --- | --- | --- | --- | --- | --- | --- | --- | --- | --- | --- | --- | --- | --- | --- | --- | --- | --- | --- | --- | --- | --- | --- | --- | --- | --- | --- | --- | --- | --- | --- | --- | --- | --- | --- | --- | --- | --- | --- | --- | --- | --- | --- | --- | --- | --- | --- | --- | --- | --- | --- | --- | --- | --- | --- | --- | --- | --- | --- | --- | --- | --- | --- | --- | --- | --- | --- | --- | --- | --- | --- | --- | --- | --- | --- | --- | --- | --- | --- | --- | --- | --- | --- | --- | --- | --- | --- | --- | --- | --- | --- | --- | --- | --- | --- | --- | --- | --- | --- | --- | --- | --- | --- | --- | --- | --- | --- | --- | --- | --- | --- | --- | --- | --- | --- | --- | --- | --- | --- | --- | --- | --- | --- | --- | --- | --- | --- | --- | --- | --- | --- | --- | --- | --- | --- | --- | --- | --- | --- | --- | --- | --- | --- | --- | --- | --- | --- | --- | --- | --- | --- | --- | --- | --- | --- | --- | --- | --- | --- | --- | --- | --- | --- | --- | --- | --- | --- | --- | --- | --- | --- | --- | --- | --- | --- | --- | --- | --- | --- | --- | --- | --- | --- | --- | --- | --- | --- | --- | --- | --- | --- | --- | --- | --- | --- | --- | --- | --- | --- | --- |

*p < .05; **p < .01; ***p < .001. HSC = Highly Sensitive Child scale; EOE = Ease of Excitation; LST = Low Sensory Threshold; AES = Aesthetic Sensitivity; cICAST = child report ISPCAN Child Abuse Screening Tool; Psy. con. = psychological control; Beh. con. = behavioural control; PHE = Positive Home Experiences; HSP = Highly Sensitive Person Scale; pICAST = parent-report ISPCAN Child Abuse Screening Tool; HIS = Human Insecurity; PTSD = maternal post-traumatic stress disorder; Depr. = Depression; MH1-MH3 = single item mental health questions.

| Table S5: Pearson correlations between community-level variables   \|  \| HSC \| EOE \| LST \| AES \| PREI \| Soc. sup. \| Bully \| Vio. vic. \| Coll. Eff. \| WE \| \| --- \| --- \| --- \| --- \| --- \| --- \| --- \| --- \| --- \| --- \| --- \| \| HSC \|  \|  \|  \|  \|  \|  \|  \|  \|  \|  \| \| EOE \| 0.84*** \|  \|  \|  \|  \|  \|  \|  \|  \|  \| \| LST \| 0.73*** \| 0.41*** \|  \|  \|  \|  \|  \|  \|  \|  \| \| AES \| 0.62*** \| 0.28*** \| 0.25*** \|  \|  \|  \|  \|  \|  \|  \| \| PREI \| 0.05* \| 0.07* \| 0.01 \| 0.03 \|  \|  \|  \|  \|  \|  \| \| Soc. sup. \| 0.01 \| -0.02 \| -0.03 \| 0.09*** \| 0.11*** \|  \|  \|  \|  \|  \| \| Bully \| 0.05 \| 0.05* \| 0.03 \| 0.01 \| -0.03 \| -0.12*** \|  \|  \|  \|  \| \| Vio. vic. \| 0.05 \| 0.04 \| 0.03 \| 0.03 \| 0.00 \| -0.11*** \| 0.59*** \|  \|  \|  \| \| Coll. Eff. \| 0.04 \| 0.02 \| 0.00 \| 0.08** \| 0.34*** \| 0.11*** \| 0.00 \| 0.00 \|  \|  \| \| WE \| 0.08** \| 0.08** \| 0.06* \| 0.02 \| -0.20*** \| -0.14*** \| 0.16*** \| 0.17*** \| -0.06* \|  \| |
| --- | --- | --- | --- | --- | --- | --- | --- | --- | --- | --- | --- | --- | --- | --- | --- | --- | --- | --- | --- | --- | --- | --- | --- | --- | --- | --- | --- | --- | --- | --- | --- | --- | --- | --- | --- | --- | --- | --- | --- | --- | --- | --- | --- | --- | --- | --- | --- | --- | --- | --- | --- | --- | --- | --- | --- | --- | --- | --- | --- | --- | --- | --- | --- | --- | --- | --- | --- | --- | --- | --- | --- | --- | --- | --- | --- | --- | --- | --- | --- | --- | --- | --- | --- | --- | --- | --- | --- | --- | --- | --- | --- | --- | --- | --- | --- | --- | --- | --- | --- | --- | --- | --- | --- | --- | --- | --- | --- | --- | --- | --- | --- | --- | --- | --- | --- | --- | --- | --- | --- | --- | --- |
| *p < .05; **p < .01; ***p < .001. HSC = Highly Sensitive Child scale; EOE = Ease of Excitation; LST = Low Sensory Threshold; AES = Aesthetic Sensitivity; PREI = Perceived Refugee Environment Index; Soc. sup = Social Support; Vio. vic. = violent victimisation; Coll. Eff. = Collective Efficacy; WE = war exposure. |

| Table S6: Global test of no regression   \|  \| Estimate \| Standard Error \| t value \| Pr(>\|t\|) \|  \| \| --- \| --- \| --- \| --- \| --- \| --- \| \| (Intercept) \| -0.904 \| 0.880 \| -1.028 \| 0.3040 \|  \| \| Child Age \| 0.054 \| 0.013 \| 4.141 \| 0.0000 \| *** \| \| Mother age \| 0.000 \| 0.005 \| 0.056 \| 0.9557 \|  \| \| Number of children \| 0.000 \| 0.016 \| 0.003 \| 0.9977 \|  \| \| Birth order of participating child \| -0.011 \| 0.019 \| -0.559 \| 0.5760 \|  \| \| Witnessing abuse \| -1.546 \| 1.227 \| -1.260 \| 0.2079 \|  \| \| Verbal Abuse \| -1.539 \| 1.227 \| -1.255 \| 0.2098 \|  \| \| Neglect \| -1.538 \| 1.228 \| -1.253 \| 0.2104 \|  \| \| Physical abuse \| -1.555 \| 1.227 \| -1.267 \| 0.2055 \|  \| \| Forced work \| -0.014 \| 0.017 \| -0.809 \| 0.4189 \|  \| \| Abuse total \| 1.556 \| 1.227 \| 1.268 \| 0.2051 \|  \| \| Psychological control \| 0.016 \| 0.011 \| 1.392 \| 0.1641 \|  \| \| Acceptance \| 0.029 \| 0.008 \| 3.820 \| 0.0001 \| *** \| \| Behavioural control \| 0.068 \| 0.014 \| 5.012 \| 0.0000 \| *** \| \| Conflict \| 0.007 \| 0.009 \| 0.782 \| 0.4342 \|  \| \| Positive Home Experiences \| 0.027 \| 0.006 \| 4.346 \| 0.0000 \| *** \| \| PREI \| 0.219 \| 0.063 \| 3.495 \| 0.0005 \| *** \| \| HSP \| 0.038 \| 0.032 \| 1.208 \| 0.2273 \|  \| \| Physical abuse caregiver \| 0.000 \| 0.004 \| 0.044 \| 0.9652 \|  \| \| Human Insecurity \| 0.294 \| 0.073 \| 4.003 \| 0.0001 \| *** \| \| PTSD \| -0.002 \| 0.002 \| -0.901 \| 0.3677 \|  \| \| Anxiety \| 0.030 \| 0.007 \| 4.401 \| 0.0000 \| *** \| \| Depression \| -0.012 \| 0.007 \| -1.697 \| 0.0899 \| . \| \| Impulsivity \| 0.017 \| 0.005 \| 3.279 \| 0.0011 \| ** \| \| MH1 \| -0.025 \| 0.041 \| -0.603 \| 0.5468 \|  \| \| MH2 \| 0.079 \| 0.044 \| 1.806 \| 0.0711 \| . \| \| MH3 \| 0.059 \| 0.028 \| 2.119 \| 0.0343 \| * \| \| Perceived stress \| -0.004 \| 0.009 \| -0.433 \| 0.6653 \|  \| \| Family support \| -0.356 \| 0.331 \| -1.076 \| 0.2823 \|  \| \| Friend support \| -0.281 \| 0.330 \| -0.851 \| 0.3947 \|  \| \| Social support \| 0.592 \| 0.660 \| 0.897 \| 0.3700 \|  \| \| Bullying \| 0.008 \| 0.007 \| 1.228 \| 0.2196 \|  \| \| Violent victimisation \| 0.017 \| 0.020 \| 0.844 \| 0.3988 \|  \| \| Informal social control \| 0.143 \| 0.412 \| 0.346 \| 0.7290 \|  \| \| Social cohesion and trust \| 0.140 \| 0.412 \| 0.339 \| 0.7348 \|  \| \| Collective Efficacy \| -0.141 \| 0.412 \| -0.342 \| 0.7324 \|  \| \| War Exposure \| 0.006 \| 0.005 \| 1.118 \| 0.2638 \|  \| \| PGS000327 \| 0.000 \| 0.007 \| 0.072 \| 0.9427 \|  \| \| PGS002790 \| -0.159 \| 0.432 \| -0.367 \| 0.7138 \|  \| \| PGS002746 \| -0.000 \| 0.000 \| -0.210 \| 0.8337 \|  \| \| PGS003753 \| -0.003 \| 0.006 \| -0.584 \| 0.5596 \|  \| \| Sex (female) \| -0.215 \| 0.055 \| -3.906 \| 0.0001 \| *** \| \| Left Syria: less than 1 year ago \| -0.138 \| 0.427 \| -0.323 \| 0.7471 \|  \| \| 1-2 years ago \| 0.033 \| 0.427 \| 0.078 \| 0.9379 \|  \| \| 2-3 years ago \| 0.041 \| 0.427 \| 0.095 \| 0.9243 \|  \| \| 3-4 years ago \| -0.035 \| 0.424 \| -0.082 \| 0.9346 \|  \| \| More than 4 years ago \| -0.071 \| 0.427 \| -0.167 \| 0.8672 \|  \| \| Maternal education: None \| 0.274 \| 0.480 \| 0.572 \| 0.5674 \|  \| \| Up to Grade 6 \| 0.300 \| 0.480 \| 0.625 \| 0.5320 \|  \| \| Up to Grade 9 \| 0.187 \| 0.487 \| 0.384 \| 0.7007 \|  \| \| Up to Grade 12 \| 0.167 \| 0.520 \| 0.321 \| 0.7480 \|  \| \| *Significance codes: 0 <= '***' < 0.001 < '**' < 0.01 < '*' < 0.05* \| \| \| \| \| \| \|  \| \| \| \| \| \| \| Residual standard error: 0.9388 on 1358 degrees of freedom \| \| \| \| \| \| \| Multiple R-squared: 0.172, Adjusted R-squared: 0.1415 \| \| \| \| \| \| \| F-statistic: 5.643 on 1358 and 50 DF, p-value: 0.0000 \| \| \| \| \| \| |
| --- | --- | --- | --- | --- | --- | --- | --- | --- | --- | --- | --- | --- | --- | --- | --- | --- | --- | --- | --- | --- | --- | --- | --- | --- | --- | --- | --- | --- | --- | --- | --- | --- | --- | --- | --- | --- | --- | --- | --- | --- | --- | --- | --- | --- | --- | --- | --- | --- | --- | --- | --- | --- | --- | --- | --- | --- | --- | --- | --- | --- | --- | --- | --- | --- | --- | --- | --- | --- | --- | --- | --- | --- | --- | --- | --- | --- | --- | --- | --- | --- | --- | --- | --- | --- | --- | --- | --- | --- | --- | --- | --- | --- | --- | --- | --- | --- | --- | --- | --- | --- | --- | --- | --- | --- | --- | --- | --- | --- | --- | --- | --- | --- | --- | --- | --- | --- | --- | --- | --- | --- | --- | --- | --- | --- | --- | --- | --- | --- | --- | --- | --- | --- | --- | --- | --- | --- | --- | --- | --- | --- | --- | --- | --- | --- | --- | --- | --- | --- | --- | --- | --- | --- | --- | --- | --- | --- | --- | --- | --- | --- | --- | --- | --- | --- | --- | --- | --- | --- | --- | --- | --- | --- | --- | --- | --- | --- | --- | --- | --- | --- | --- | --- | --- | --- | --- | --- | --- | --- | --- | --- | --- | --- | --- | --- | --- | --- | --- | --- | --- | --- | --- | --- | --- | --- | --- | --- | --- | --- | --- | --- | --- | --- | --- | --- | --- | --- | --- | --- | --- | --- | --- | --- | --- | --- | --- | --- | --- | --- | --- | --- | --- | --- | --- | --- | --- | --- | --- | --- | --- | --- | --- | --- | --- | --- | --- | --- | --- | --- | --- | --- | --- | --- | --- | --- | --- | --- | --- | --- | --- | --- | --- | --- | --- | --- | --- | --- | --- | --- | --- | --- | --- | --- | --- | --- | --- | --- | --- | --- | --- | --- | --- | --- | --- | --- | --- | --- | --- | --- | --- | --- | --- | --- | --- | --- | --- | --- | --- | --- | --- | --- | --- | --- | --- | --- | --- | --- | --- | --- | --- | --- | --- | --- | --- | --- | --- | --- | --- | --- | --- | --- | --- | --- | --- | --- | --- | --- | --- | --- | --- | --- | --- | --- | --- | --- | --- | --- | --- | --- | --- | --- | --- | --- |

| Table S7: Predictor importance for children younger than 10   \| Variables \| Number of model subsets \| GCV \| RSS \| Coefficient \| Value \| \| --- \| --- \| --- \| --- \| --- \| --- \| \| Acceptance \| 9 \| 100.00 \| 100.00 \| h(Acceptance-20) \| 0.05 \| \| Anxiety \| 8 \| 84.40 \| 89.52 \| h(Anxiety-8) \| 0.06 \| \| Positive Home Experiences \| 7 \| 73.78 \| 81.30 \| h(PHE-22) \| 0.04 \| \| Physical abuse \| 6 \| 64.07 \| 73.34 \| h(Physical abuse-3) \| 0.02 \| \| PTSD \| 5 \| 54.20 \| 65.13 \| h(33-PTSD) \| 0.02 \| \| Child Age \| 4 \| 40.10 \| 55.17 \| h(8.84873-Age) \| -0.27 \| \| PREI \| 2 \| 25.42 \| 37.94 \| h(3.25327-PREI) \| -0.37 \| \| Impulsivity \| 2 \| 25.42 \| 37.94 \| h(24-Impulsivity) \| -0.04 \| \| PGS002746 \| 2 \| 18.99 \| 36.32 \| h(PGS002746-225.428) \| -0.001 \| \| Intercept \|  \|  \|  \|  \| 4.42 \| |
| --- | --- | --- | --- | --- | --- | --- | --- | --- | --- | --- | --- | --- | --- | --- | --- | --- | --- | --- | --- | --- | --- | --- | --- | --- | --- | --- | --- | --- | --- | --- | --- | --- | --- | --- | --- | --- | --- | --- | --- | --- | --- | --- | --- | --- | --- | --- | --- | --- | --- | --- | --- | --- | --- | --- | --- | --- | --- | --- | --- | --- | --- | --- | --- | --- | --- | --- |

GCV – generalized cross-validation; RSS – residual sum of squares

| Table S8: Predictor importance for children between 10-12 years old   \| Variables \| Number of model subsets \| GCV \| RSS \| Coefficient \| Value \| \| --- \| --- \| --- \| --- \| --- \| --- \| \| Behavioural control \| 2 \| 100.00 \| 100.00 \|  \| 0.16 \| \| Human Insecurity \| 1 \| 55.15 \| 56.98 \| h(Insecurity-3.4) \| 1.05 \| \| Intercept \|  \|  \|  \|  \| 2.45 \| |
| --- | --- | --- | --- | --- | --- | --- | --- | --- | --- | --- | --- | --- | --- | --- | --- | --- | --- | --- | --- | --- | --- | --- | --- | --- |

GCV – generalized cross-validation; RSS – residual sum of squares; h - hinge

| Table S9: Predictor importance for children aged 13 and older   \| Variables \| Number of model subsets \| GCV \| RSS \| Coefficient \| Value \| \| --- \| --- \| --- \| --- \| --- \| --- \| \| Anxiety \| 10 \| 100.00 \| 100.000 \| h(Anxiety-8) \| 0.08 \| \| Positive Home Experiences \| 9 \| 81.46 \| 85.15 \| h(PHE-21) \| 0.07 \| \| MH3 \| 8 \| 69.47 \| 75.12 \| h(2-MH3) \| -0.35 \| \| Child Age \| 7 \| 59.35 \| 66.44 \| h(Child Age-13.7714) \| 0.17 \| \| Neglect \| 5 \| 37.74 \| 48.65 \|  \| 0.05 \| \| Sex (male) \| 5 \| 37.74 \| 48.65 \|  \| -0.30 \| \| PREI \| 2 \| 21.42 \| 29.27 \| h(PREI-3.21057) \| 0.49 \| \| Impulsivity \| 2 \| 21.42 \| 29.27 \| h(Impulsivity-24): \| 0.04 \| \| Time since leaving Syria (3-4 years ago) \| 2 \| 21.42 \| 29.27 \|  \| 0.26 \| \| PGS003753 \| 2 \| 18.41 \| 27.96 \| h(PGS003753-9.12219): \| -0.03 \| \| Intercept \|  \|  \|  \|  \| 4.69 \| \|  \|  \|  \|  \|  \|  \| |
| --- | --- | --- | --- | --- | --- | --- | --- | --- | --- | --- | --- | --- | --- | --- | --- | --- | --- | --- | --- | --- | --- | --- | --- | --- | --- | --- | --- | --- | --- | --- | --- | --- | --- | --- | --- | --- | --- | --- | --- | --- | --- | --- | --- | --- | --- | --- | --- | --- | --- | --- | --- | --- | --- | --- | --- | --- | --- | --- | --- | --- | --- | --- | --- | --- | --- | --- | --- | --- | --- | --- | --- | --- | --- | --- | --- | --- | --- | --- |

GCV – generalized cross-validation; RSS – residual sum of squares

| Table S10: Predictor importance for boys   \| Variables \| Number of model subsets \| GCV \| RSS \| Coefficient \| Value \| \| --- \| --- \| --- \| --- \| --- \| --- \| \| Positive Home Experiences \| 10 \| 100.00 \| 100.00 \| h(PHE-22) \| 0.07 \| \| Behavioural control \| 9 \| 80.13 \| 84.42 \|  \| 0.08 \| \| Verbal Abuse \| 7 \| 66.37 \| 71.40 \| h(2-Verbal Abuse) \| -0.11 \| \| Human Insecurity \| 7 \| 58.82 \| 66.45 \| h(Insecurity-3.33333) \| 0.54 \| \| Psychological control \| 6 \| 49.66 \| 58.49 \| h(Control-10) \| 0.06 \| \| Neglect \| 5 \| 39.64 \| 50.06 \|  \| 0.05 \| \| PREI \| 5 \| 39.64 \| 50.06 \| h(PREI-3.26458) \| 0.50 \| \| Physical abuse (caregiver-report) \| 3 \| 30.18 \| 38.33 \| h(9-Abuse) \| 0.04 \| \| Anxiety \| 2 \| 23.03 \| 30.39 \| h(Anxiety-7) \| 0.03 \| \| MH3 \| 1 \| 14.95 \| 20.79 \| h(MH3-2) \| 0.15 \| \| Intercept \|  \|  \|  \|  \| 3.02 \| |
| --- | --- | --- | --- | --- | --- | --- | --- | --- | --- | --- | --- | --- | --- | --- | --- | --- | --- | --- | --- | --- | --- | --- | --- | --- | --- | --- | --- | --- | --- | --- | --- | --- | --- | --- | --- | --- | --- | --- | --- | --- | --- | --- | --- | --- | --- | --- | --- | --- | --- | --- | --- | --- | --- | --- | --- | --- | --- | --- | --- | --- | --- | --- | --- | --- | --- | --- | --- | --- | --- | --- | --- | --- |

GCV – generalized cross-validation; RSS – residual sum of squares

| Table S11: Predictor importance for girls   \| Variables \| Number of model subsets \| GCV \| RSS \| Coefficient \| Value \| \| --- \| --- \| --- \| --- \| --- \| --- \| \| Anxiety \| 9 \| 100.00 \| 100.00 \| h(Anxiety-8) \| 0.05 \| \| Child Age \| 9 \| 90.14 \| 93.97 \| h(Child_Age-10.8775) \| 0.09 \| \| PREI \| 8 \| 79.83 \| 85.52 \| h(3.19405-PREI) \| -0.52 \| \| War Exposure \| 7 \| 72.48 \| 78.63 \| h(War_Exposure-9) \| 0.03 \| \| Acceptance \| 6 \| 66.51 \| 72.33 \| h(Acceptance-19) \| 0.03 \| \| Impulsivity \| 6 \| 66.51 \| 72.33 \| h(Impulsivity-24) \| 0.05 \| \| PTSD \| 4 \| 41.85 \| 52.67 \| h(34-PTSD) \| 0.01 \| \| Abuse (total) \| 2 \| 22.16 \| 34.06 \| h(Abuse-8) \| 0.01 \| \| Positive Home Experiences \| 2 \| 22.16 \| 34.06 \| h(PHE-21.6) \| 0.03 \| \| Human Insecurity \| 1 \| 12.89 \| 23.11 \| h(Insecurity-3.33333) \| 0.35 \| \| Intercept \|  \|  \|  \|  \| 4.14 \| |
| --- | --- | --- | --- | --- | --- | --- | --- | --- | --- | --- | --- | --- | --- | --- | --- | --- | --- | --- | --- | --- | --- | --- | --- | --- | --- | --- | --- | --- | --- | --- | --- | --- | --- | --- | --- | --- | --- | --- | --- | --- | --- | --- | --- | --- | --- | --- | --- | --- | --- | --- | --- | --- | --- | --- | --- | --- | --- | --- | --- | --- | --- | --- | --- | --- | --- | --- | --- | --- | --- | --- | --- | --- |

GCV – generalized cross-validation; RSS – residual sum of squares

| Table S12: Variables selected via stepwise selection   \| **Characteristic** \| **Beta** \| **95% CI**^1^ \| **p-value** \| \| --- \| --- \| --- \| --- \| \| Child age \| 0.05 \| 0.03, 0.08 \| <0.001 \| \| Abuse (child report) \| 0.01 \| 0.00, 0.02 \| <0.001 \| \| Maternal acceptance \| 0.02 \| 0.01, 0.04 \| 0.002 \| \| Behavioural control \| 0.07 \| 0.04, 0.10 \| <0.001 \| \| PHE \| 0.03 \| 0.02, 0.04 \| <0.001 \| \| PREI \| 0.29 \| 0.17, 0.41 \| <0.001 \| \| Human insecurity \| 0.34 \| 0.19, 0.49 \| <0.001 \| \| Maternal anxiety \| 0.03 \| 0.02, 0.05 \| <0.001 \| \| Maternal depression \| -0.02 \| -0.03, -0.01 \| 0.002 \| \| Maternal impulsivity \| 0.02 \| 0.01, 0.03 \| <0.001 \| \| MH3 \| 0.09 \| 0.03, 0.14 \| 0.003 \| \| Family support \| -0.08 \| -0.15, -0.02 \| 0.016 \| \| Bullying \| 0.01 \| 0.00, 0.02 \| 0.042 \| \| Sex (male) \| -0.19 \| -0.30, -0.07 \| 0.001 \| \| Leaving Syria 12-24 months ago \| 0.18 \| 0.02, 0.33 \| 0.024 \| \| ^1^CI = Confidence Interval; PHE = Positive Home Experiences; PREI = Perceived Refugee Environment Index; MH3 = single mental health item 3. \| \| \| \| |
| --- | --- | --- | --- | --- | --- | --- | --- | --- | --- | --- | --- | --- | --- | --- | --- | --- | --- | --- | --- | --- | --- | --- | --- | --- | --- | --- | --- | --- | --- | --- | --- | --- | --- | --- | --- | --- | --- | --- | --- | --- | --- | --- | --- | --- | --- | --- | --- | --- | --- | --- | --- | --- | --- | --- | --- | --- | --- | --- | --- | --- | --- | --- | --- | --- | --- | --- | --- | --- |

| Table S13: Variables selected via penalised regression   \|  \| Model Coefficients \| \| \| --- \| --- \| --- \| \| Variable \| LASSO \| Elastic net \| \| Abuse (child report) \| 0.003 \| 0.002 \| \| Behavioural control \| 0.058 \| 0.063 \| \| Bullying \| 0.001 \| 0.007 \| \| Child age \| 0.023 \| 0.037 \| \| Human insecurity \| 0.155 \| 0.244 \| \| Intercept \| 2.033 \| 0.529 \| \| Left Syria 12-24 months ago \|  \| 0.095 \| \| Left Syria 24-36 months ago \|  \| 0.072 \| \| Maternal acceptance \| 0.003 \| 0.018 \| \| Maternal anxiety \| 0.015 \| 0.021 \| \| Maternal impulsivity \| 0.006 \| 0.014 \| \| MH3 \| 0.035 \| 0.065 \| \| Neglect (child report) \| 0.013 \| 0.018 \| \| Parent-child conflict \| 0.000 \| 0.005 \| \| PHE \| 0.021 \| 0.024 \| \| PREI \| 0.118 \| 0.194 \| \| Psychological control \| 0.001 \| 0.010 \| \| Verbal abuse (child report) \| 0.006 \| 0.012 \| \| War Exposure \|  \| 0.004 \| |
| --- | --- | --- | --- | --- | --- | --- | --- | --- | --- | --- | --- | --- | --- | --- | --- | --- | --- | --- | --- | --- | --- | --- | --- | --- | --- | --- | --- | --- | --- | --- | --- | --- | --- | --- | --- | --- | --- | --- | --- | --- | --- | --- | --- | --- | --- | --- | --- | --- | --- | --- | --- | --- | --- | --- | --- | --- | --- | --- | --- | --- | --- | --- | --- |

PHE = Positive Home Experiences; PREI = Perceived Refugee Environment Index

*Table S14: Predictors of residual difference in sensitivity from wave 1 to wave 2*

| Variables | Number of model subsets | GCV | RSS | Coefficient | Value |
| --- | --- | --- | --- | --- | --- |
| Abuse (verbal; wave 2) | 12 | 100.00 | 100.00 |  | 0.06 |
| Sex (female) | 11 | 86.13 | 89.65 |  | 0.31 |
| Positive Home Experiences (wave 2) | 10 | 69.08 | 77.79 | h(22-PHE) | -0.05 |
| HSC (wave 1) | 9 | 59.61 | 70.49 | h(4.91667-HSC) | -0.16 |
| Abuse (physical; wave 1) | 8 | 52.27 | 64.34 | h(Abuse-3) | 0.02 |
| PREI (wave 2) | 7 | 46.70 | 59.02 | h(PREI-3.31633)  h(3.31633-PREI) | -0.40  -0.45 |
| Support (family; wave 2) | 5 | 41.16 | 50.55 | h(Support-5.6): | 0.16 |
| Number of children | 4 | 32.97 | 43.32 | h(Number-6) | -0.05 |
| HSP (mother; wave 2) | 3 | 26.19 | 36.41 | h(HSP-5.18756) | 0.19 |
| Behavioural control (wave 2) | 2 | 18.57 | 28.54 |  | 0.05 |
| Support (family; wave 1) | 1 | 2.81 | 17.58 | h(Support-5.6) | 0.11 |
| Intercept |  |  |  |  | 3.83 |
|  |  |  |  |  |  |

GCV – generalized cross-validation; RSS – residual sum of squares; h - hinge


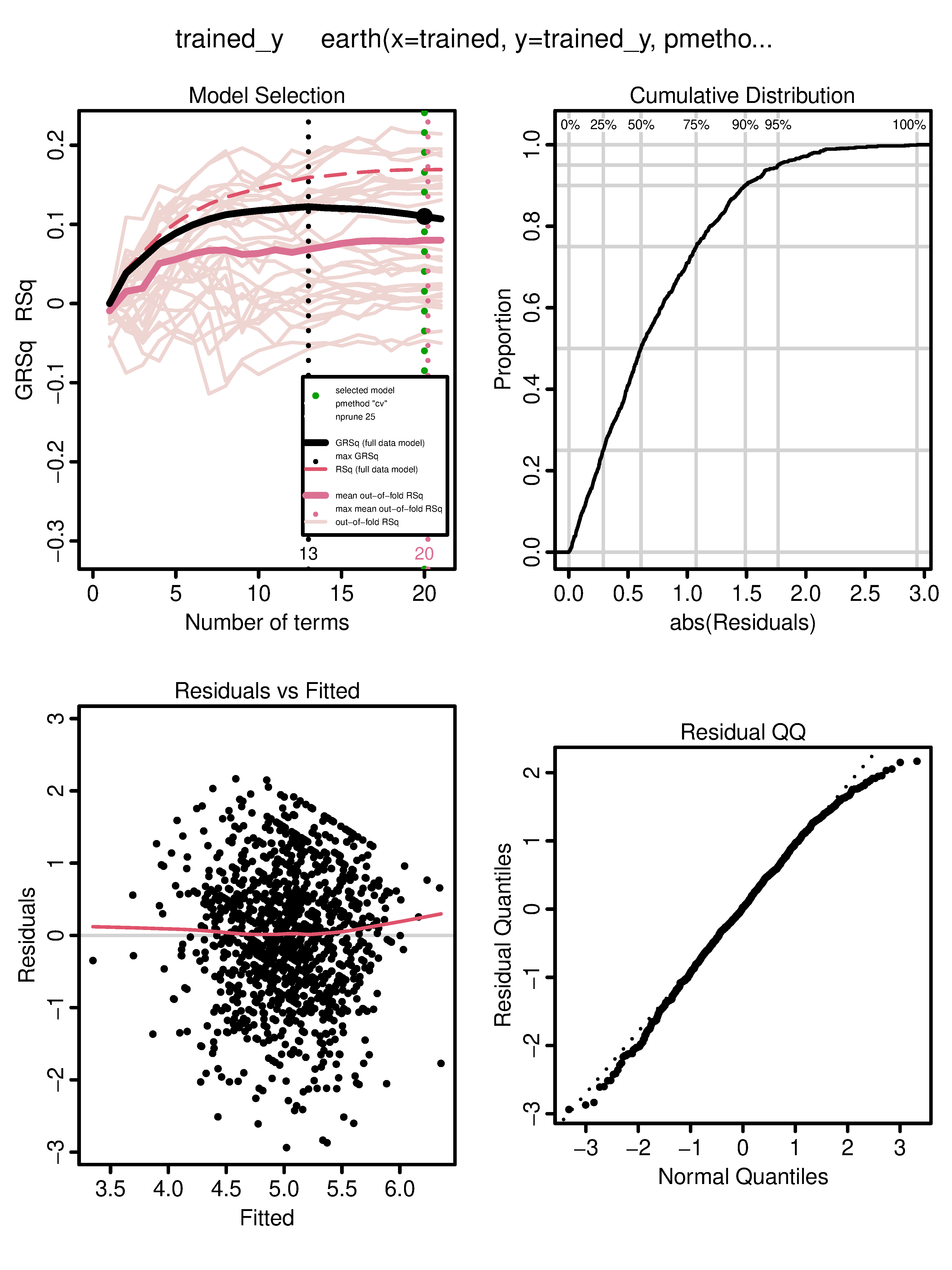


**Figure S1: MARS model diagnostics**. Diagnostic plots for the MARS model of total environmental sensitivity regressed against other study variables using 10-fold cross-validation repeated thrice.
